# The ABCG2 multidrug transporter is a pump gated by a valve and an extracellular lid

**DOI:** 10.1038/s41467-019-13302-2

**Published:** 2019-11-28

**Authors:** Narakorn Khunweeraphong, Daniel Szöllősi, Thomas Stockner, Karl Kuchler

**Affiliations:** 1 Max Perutz Labs Vienna, Center for Medical Biochemistry, Medical University of Vienna, Campus Vienna Biocenter, Dr. Bohr-Gasse 9/2, 1030 Vienna, Austria; 2 Center for Physiology and Pharmacology, Institute of Pharmacology, Medical University of Vienna, Währingerstrasse 13A, 1090 Vienna, Austria

**Keywords:** Biochemistry, Biological techniques, Biophysics, Cancer, Cell biology

## Abstract

The human ATP-binding cassette transporter ABCG2 is a key to anticancer resistance and physiological detoxification. However, the molecular mechanism of substrate transport remains enigmatic. A hydrophobic di-leucine motif in the ABCG2 core separates a large intracellular cavity from a smaller upper cavity. We show that the di-leucine motif acts as a valve that controls drug extrusion. Moreover, the extracellular structure engages the re-entry helix and all extracellular loops to form a roof architecture on top of the upper cavity. Disulfide bridges and a salt bridge limit roof flexibility, but provide a lid-like function to control drug release. We propose that drug translocation from the central to the upper cavities through the valve is driven by a squeezing motion, suggesting that ABCG2 operates similar to a peristaltic pump. Finally, the roof contains essential residues, offering therapeutic options to block ABCG2 by either targeting the valve or essential residues in the roof.

## Abstract (web summary)

The human ATP-binding cassette transporter ABCG2 plays critical roles in anticancer resistance but the molecular mechanism of ABCG2-mediated substrate transport remains enigmatic. Here authors use extensive mutagenesis and molecular dynamics simulations to reveal a mechanistic basis for the function of the di-leucine valve and the roof organization in the transport cycle.

## Introduction

ATP-binding cassette (ABC) transporters are dedicated transport proteins that mediate translocation of diverse physiological and xenobiotic substrates across cellular membranes in an ATP-dependent manner^1,2^. Seven human ABC protein subfamilies, A to G^3^, are known. ABCG2, also known as breast cancer resistance protein (BCRP), is a half transporter that plays an essential role in physiological detoxification across tissue barriers, including the placental, mammary epithelium or blood–brain barrier^4,5^. In addition, ABCG2 and the related ABC transporters P-glycoprotein (ABCB1) and MRP1 (ABCC1)^6–8^ act as brothers in arms in multidrug resistance phenomena in cancer^9^. Of note, mammalian ABCG family members share high evolutionary conservation with the fungal pleiotropic drug resistance (PDR) transporters, suggesting that they may utilize related mechanisms for substrate transport, including the coupling of the catalytic cycle with drug release^10,11^.

The first X-ray crystal structure of the dimeric ABCG5/G8 human ABCG transporter in the inward-facing state revealed a novel compact fold not seen before in any other eukaryotic ABC exporters. In fact, ABCG5/G8 resembles an importer rather than an exporter, with a central cavity, a unique assembly of putative transmembrane helices, and the polar relay^12,13^. Prominent elements at both membrane interfaces include the elbow helix at the inner membrane leaflet, and the re-entry helix embedded in the outer membrane leaflet, respectively^13^. Cryo-electron microscopy (cryo-EM) structures of human ABCG2 revealed the first structure of human ABCG2^14^, with structural coordinates that are almost identical to the ABCG5/G8 crystal structure^13^. Several independent reports model and predict essentially identical atomic structures of ABCG2, and suggest a conserved, but new fold adopted by the ABCG transporter family^15–17^. Based on a validated homology model, we have recently delineated the essential role of the intracellular transmission interface in ABCG2-mediated drug extrusion^18^, which has been confirmed by molecular dynamics (MD) simulations and docking studies^16^. Furthermore, several membrane-spanning helices contribute to substrate specificity and recognition, but also impact the conformational dynamics^17^, all in all strongly supporting the new fold present in the ABCG family^13–20^. The related fold of both ABCG5/G8 and ABCG2 also implies related transport mechanisms, hence suggesting that subfamily-specific catalytic cycles may exist^20–23^.

The ABCG2 structure contains two apparent cavities. The architecture of the central cavity includes the intracellular loop 1, the elbow helix, and residues facing the cavity from transmembrane helices and the NBD dimer. The central cavity is part of the transmission interface, which is absolutely essential for ABCG2 biogenesis and drug transport^18^. The smaller upper cavity is part of the extracellular polar roof, but its role has not been fully elucidated. Remarkably, the central and upper cavities of ABCG2^14,18–20^ are separated by two leucines, L554 and L555, facing their equivalent residues in close proximity in the core of the symmetric ABCG2 dimer. This structural element supports the notion that the ABCG2 transport cycle may also engage a gating mechanism that controls substrate movement from the central to the upper cavity along the substrate translocation pathway^24–26^. Of note, the fungal CmABCB1 transporter, although structurally very different from ABCG2, may also harbor two gates involved in substrate movement^27,28^. The CmABCB1 outer gate, which is somewhat reminiscent with the upper cavity in ABCG2, locates close the outer membrane leaflet in the outward-open state to allow for substrate translocation and/or release^27^. The aromatic hydrophobic cluster above the inner chamber is gating substrate translocation to the extracellular space^27,28^. Interestingly, the extracellular roof adopts a tightly packed lid-like architecture, which surrounds the upper transporter cavity. In addition, the roof is stabilized by intra- and intermolecular disulfide bonds connecting cysteines C592 and C608^29^, and both half transporters are linked via the C603 residue^30,31^.

Here, we have scrutinized the roof and the valve domain of ABCG2 by extensive mutagenesis and MD simulations. We reveal a mechanistic basis for the function of the di-leucine valve and the roof organization in the transport cycle. We identify critical residues within the extracellular roof that contains residues essential for both folding and ABCG2 function. The re-entry helix increases stability, and limits conformational flexibility by forming a salt bridge with the extracellular loop 1 (ECL1). Importantly, the di-leucine motif on top of the central cavity is a critical regulatory valve that acts as a hydrophobic seal in the putative translocation pathway, and thus determines substrate access to the upper cavity. Therefore, substrate translocation by ABCG2 engages at least two critical steps. First, the di-leucine valve regulates drug extrusion to the upper cavity, and is a key element of the coupling mechanism in the catalytic cycle. Second, the re-entry helix in the roof harbors the essential residues E585, which appears to be accessible from the extracellular space. This work provides fundamental mechanistic insights about how ABCG family transporters engage their extracellular architecture during drug release. The data suggest new therapeutic intervention strategies to modulate ABCG2 function in therapeutic settings by targeting either the valve or the extracellular lid.

## Results

### The ABCG2 roof is flexible and contains critical residues

**Fig. 1:**
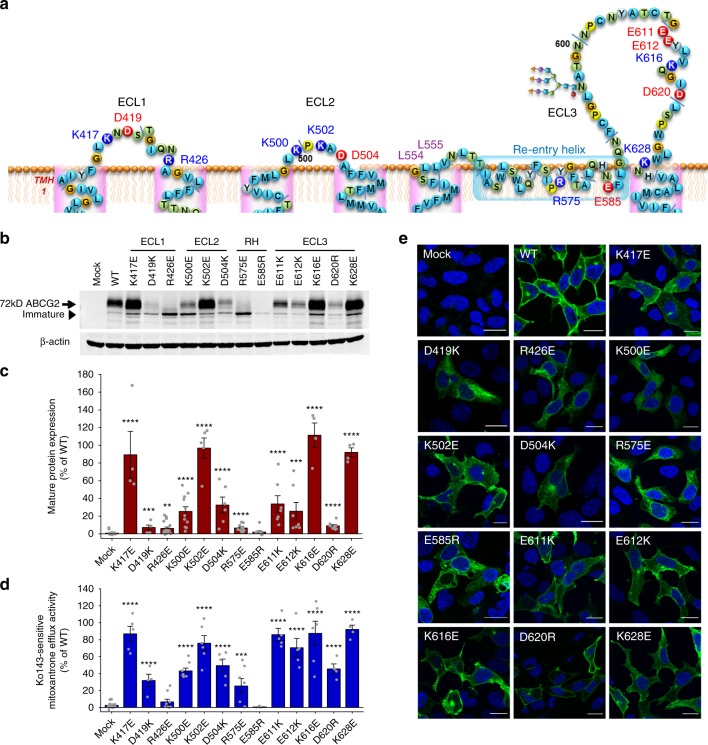
Charged residues in the extracellular roof are critical for ABCG2 function. Mutational analysis of residues in the putative extracellular membrane interface. Charged residues located in the ECL1, ECL2, re-entry helix, and ECL3 were subjected to mutational analysis: **a** Predicted membrane topology of extracellular regions in the human ABCG2 transporter. Re-entry helix is highlighted in light blue box; consensus glycosylation site is at N596. Residues subjected to mutagenesis are indicated; positively charged residues (blue); negatively charged residues (red). **b** Immunodetection of ABCG2 mutant variants using the monoclonal anti-ABCG2 (BXP-21) antibody. Mature wild-type (WT) protein migrates at ~72 kDa, the immature unglycosylated bands migrate just below. β-Actin was used as an internal loading control. **c** Odyssey-based quantification of immunoblots from several independent experiments (*n* = 3–15). Mature ABCG2 and β-actin bands are shown, and ABCG2 signals were individually normalized to β-actin and represented as the percentage of WT control. **d** Mitoxantrone efflux is shown for HEK293 cells expressing ABCG2 variants after incubation at 37 °C for 20 min in the presence and absence of the ABCG2 inhibitor Ko143. Ko143-sensitive mitoxantrone efflux is represented as percentage relative to the WT control. Data are from several independent experiments (*n* = 3–9). All data are shown as means ± SEM. ****P < 0.0001; ***P < 0.001; **P < 0.01; *P < 0.1 vs. empty plasmid-transfected HEK293 control (mock). Gray dots refer to values from each independent experiment. **e** Membrane localization of GFP-tagged ABCG2 variants. GFP signals of GFP-ABCG2 (green) were recorded in a confocal microscopy as described in Methods. DAPI was used to stain the nuclei (blue). Microscopy data are from duplicate experiments. Scale bars in microscopy images correspond to 20 µm.

We used the structural coordinates from all known cryo-EM particle structures of ABCG2^14,19,20^ and our own model^18^, to generate a refined structure of human ABCG2, emphasizing on the roof architecture (Supplementary Fig. 1). The roof organization engages ECL1, ECL2, the short linker after transmembrane helix 5 (TMH5), and the re-entry helix, as well as the much larger ECL3 (Supplementary Fig. 1a, b). Interestingly, the kinked re-entry helix engages every extracellular domain, suggesting that it stabilizes the polar roof. Hence, the extracellular roof has a compact semi-closed architecture that may act as a barrier or lid for substrate release from the upper cavity (Supplementary Fig. 1c, d). Drug release requires a conformational switch from the inward-facing to the outward-facing state. The inward-facing state of ABCG2 predicts two barriers that could control drug release. First, the extracellular roof covering and capping the upper cavity, and second, a valve-like structure residing at the top of central cavity in the middle of the Transmembrane Domain (TMDs) (Supplementary Fig. 1c). To test the functional relevance of roof residues, we subjected all extracellular charged residues to extensive mutational analysis. We generated variants of the 13 charged residues (K417, D419, R426, K500, K502, D504, R575, E585, E611, E612, K616, D620, and K628) of the roof (Fig. 1a), including GFP-ABCG2 versions of all mutants. ABCG2 variants were expressed in HEK293 cells for further functional testing, including quantification of protein levels, transport activity, ATPase activity, and membrane targeting. Charge-reversal mutations of K417, K502, K616, and K628 had little or no impact on ABCG2 function (Fig. 1b–e). However, changes of the two highly conserved ECL1 residues D419 and R426 into D419K and R426E strongly reduced mature protein levels, impaired membrane targeting, and mitoxantrone efflux. Of note, K500 and D504 are not conserved, but the charge-reversal mutants of K500E and D504K reduced mature surface protein levels and debilitated ABCG2 transport activity. Interestingly, mutational change of the highly conserved R575 and E585 residues in the re-entry helix to R575E and E585R abolished mature ABCG2 levels and efflux function, as well as proper surface trafficking as evident from the intracellular accumulation. The changes of the conserved E611, E612, and D620 residues in ECL3 into E611K, E612K, and D620R reduced protein levels, but only mildly affected mitoxantrone efflux (Fig. 1b–e). These results show that each putative ECL contains at least one charged residue required for ABCG2 function (Fig. 1b–e and Supplementary Fig. 2).

**Fig. 2:**
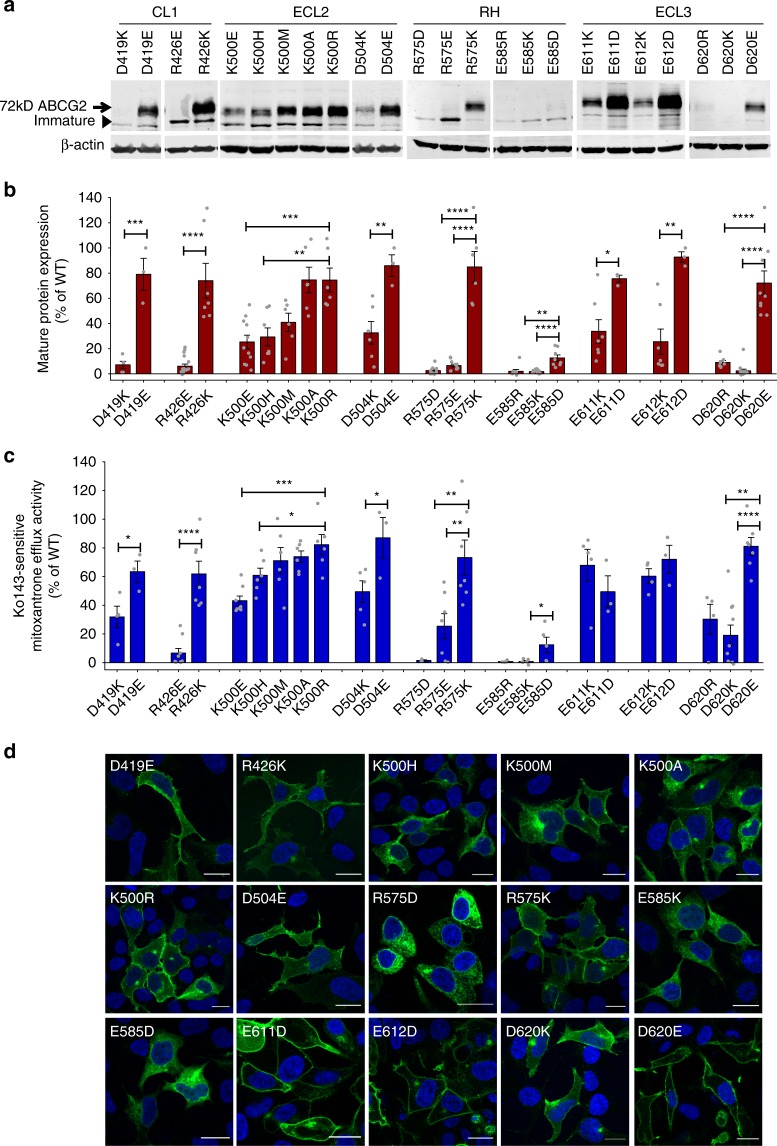
Functional analysis of the extracellular ABCG2 residues. Residues in the extracellular ABCG2 regions were subjected to mutational analysis, followed by functional expression in HEK293 cells. **a** Immunodetection of ABCG2 variants after transient transfection into HEK293 cells for 2 days is shown. β-Actin was used as a loading control. **b** Odyssey-based quantification of immunoblots from several independent experiments (*n* = 3–14). ABCG2 signals were individually normalized to β-actin and shown as the percentage of the WT control. Cell-free extracts from HEK293 cells were prepared from the same experiment; immunoblots from different gels are separated by white spaces. **c** Mitoxantrone efflux is shown for HEK293 cells transfected with ABCG2 variants. Mitoxantrone accumulation was quantified in the presence and absence of Ko143 inhibitor after incubation for 20 min at 37 °C. Ko143-sensitive mitoxantrone efflux is given as percentage efflux activity relative to WT control. Data are from several independent experiments (*n* = 3–9). All results are represented as means ± SEM; ****P < 0.0001; ***P < 0.001; **P < 0.01; *P < 0.1. Gray dots represent values from each independent experiment. **d** Membrane localization of GFP-tagged ABCG2 mutants was detected by fluorescence microscopy in the GFP channel (green) as described in Methods. Nuclei were stained with DAPI (blue). Microscopy data are from representative duplicate experiments. Scale bars in microscopy images correspond to 20 µm.

To confirm the importance of these residues, we systematically generated both conservative and charge modification mutants (Fig. 2). Notably, most conservative mutations (D419E, R426K, K500R, D504E, R575K, E611D, E612D, and D620E) maintained mature surface ABCG2 levels comparable to the wild-type (WT) control, with only minor impacts on efflux function (Fig. 2a–d). However, any changes of E585 in the re-entry helix to E585R/K/D abrogated protein levels, membrane targeting, and mitoxantrone efflux (Fig. 2).

**Fig. 3:**
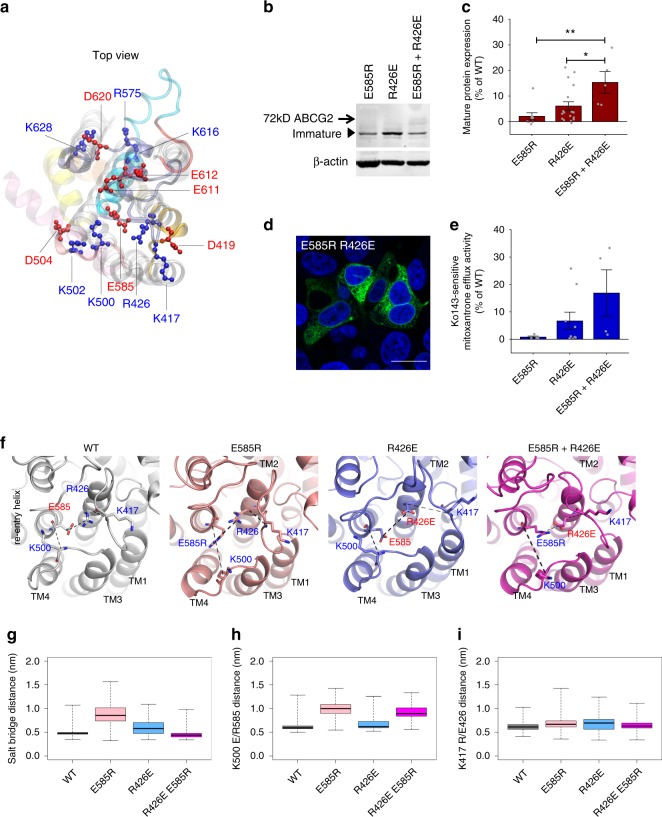
Salt bridge interaction of charged residues stabilizes extracellular roof architecture. Charged residues in extracellular domains were mutated and transiently expressed in HEK293 cells. **a** Zoom-in top view shows side chains as ball-and-stick with the positions of negative (red) and positive charges (blue) in the roof. For clarity, ECL1 (gold), ECL2 (old rose), short loop after TMH5 (red), re-entry helix (cyan), ECL3 (violet), TMHs (gray), elbow helix (pink); the intracellular NBD was removed. **b** Immunoblot detection is shown for ABCG2 mutants using the anti-ABCG2 (BXP-21) antibody in HEK293 cell-free extracts. β-Actin served as an internal loading control. **c** Quantification of immunoblot is shown for ABCG2 variants (*n* = 5–17). Data reflect fold-change relative to the WT control. All values are means ± SEM; **P < 0.01; *P < 0.1. Gray dots represent independent biological replicates. **d** Intracellular localization of the GFP-tagged ABCG2 double mutant R426E E585R in HEK293 cells. The GFP-ABCG2 signal (green) and nuclei (blue) were detected using the Zeiss LSM700 confocal microscope. Scale bar in microscopy images corresponds to 20 µm. **e** Mitoxantrone efflux activity of ABCG2 mutants expressed in HEK293 cells are shown as percentage relative to WT control (*n* = 4–9). Gray refer to independent biological replicates. **f** Conformation of WT, E585R, R426E, and double mutants, taken from the final frames of MD simulations of inward-facing ABCG2, highlighting residues 426 and 585 and the interacting residues on ECL1 (K417) and ECL2 (K500). Dotted lines indicate distances. **g** Boxplot of distances measured between Cδ of residue 585 and Cζ of residue 426 (boxes indicate second and third quantiles; the center line shows the mean, the dotted whiskers reach the extremes). **h** Distance boxplot observed between the Cα of residues E585 of the re-entry helix and K500 of ECL2. **i** Distance boxplot between residue R426 and K417 on ECL1. Data were obtained and merged from three parallel independent simulations over 150 ns for mutants, 500 ns for WT, and shown for both ABCG2 halves at 1 ns space of sampling. The data in **g**–**i** were computed based on more than 9000 data points.

The roof structure places residue R426 in ECL1 in close proximity to E585 in the re-entry helix such that they could potentially form salt bridges (Fig. 3a). To test whether ABCG2 forms a salt bridge at the extracellular region to stabilize the structure, we tested a possible interaction of the highly conserved residues R426 and E585 (Supplementary Fig. 2). The single mutants R426E or E585R showed completely abolished protein expression, mitoxantrone efflux, and membrane localization (Fig. 1b–e). Strikingly, however, the double mutant R426E E585R partially and significantly restored ABCG2 expression (Fig. 3b, c) owing to the partial restoration of the salt bridge. However, no significant membrane surface localization and mitoxantrone efflux activity was observed, most likely because E585 does not tolerate any mutational changes without losing efflux function, and because a glutamic acid rather than aspartic acid is required at position 585. Thus, residue E585, which is highly conserved in both ABCGs and yeast PDRs, is invariable for ABCG2 biogenesis and function.

We then conducted MD simulations to verify this salt bridge between ECL1 and the re-entry helix. Indeed, a salt bridge was present in WT and in the charge-reversal double mutant of E585R R426E. In contrast, the E585R and R426E single mutants altered their side chain orientation and changed the structure of the polar roof. Thus, both residues form a central hub connecting TMH1, 2, 3, 4 and the re-entry helix. This explained the only partial recovery of ABCG2 levels and function in the E585R R426E double mutant (Fig. 3f, g). The E585R mutant also disrupted an interaction of the re-entry helix with the backbone of ECL2, which thus repositioned as a consequence of this disengagement (Fig. 3h), while the side chain of R426 restrained the position of ECL1 (Fig. 3i) by a hydrogen bond towards the backbone of K417 on ECL1 (Fig. 3f). Additional interactions appeared to connect ECL1 and ECL2 (Fig. 3f). The single mutants E585R and R426E had also long-range effects, leading to an overall altered stability of the upper cavity.

### The valve is essential for drug access to the upper cavity

**Fig. 4:**
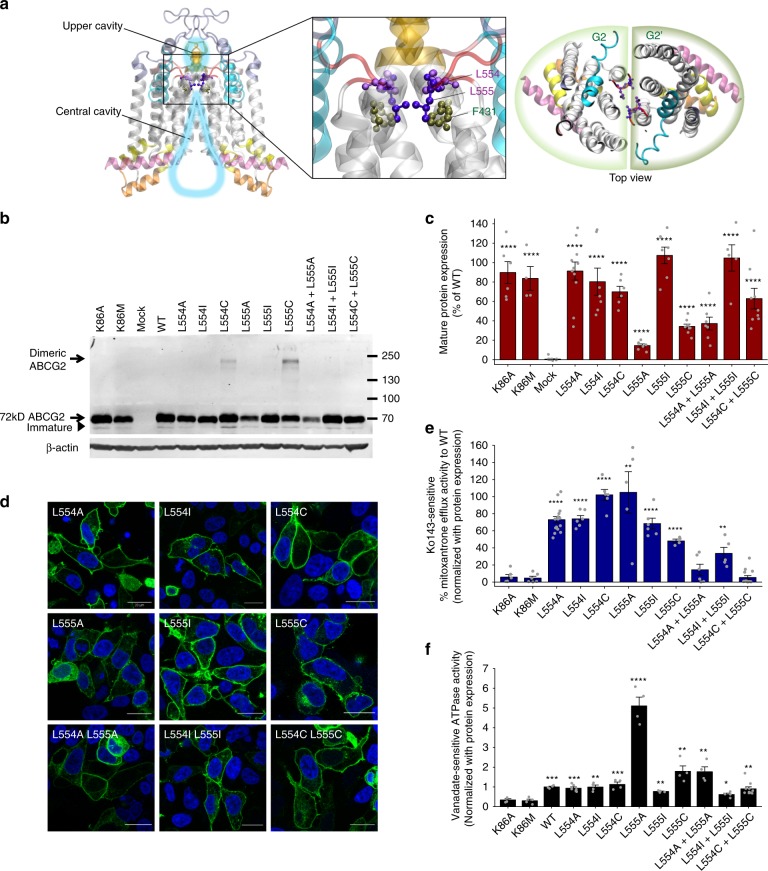
The di-leucine motif at the top of the central cavity is critical for ABCG2 function. **a** Ribbon representation of homo-dimeric ABCG2 in the ATP-free state. The zoom-in view shows the positions of L554 and L555 (purple balls-and-sticks) subtending the extracellular membrane border between the central and upper cavities. F431 (dark green balls-and-sticks) locates just below the di-leucine valve. **b** Immunodetection is shown for ABCG2 variants stably expressed in HEK293 cells and detected by the anti-ABCG2 (BXP-21) antibody. ABCG2 monomers migrate at ~72 kDa, while dimeric ABCG2 is located above. β-Actin was used as an internal loading control. **c** Odyssey-based quantification of stably expressed ABCG2 variant, normalized against WT ABCG2, is plotted. Data are from several independent experiments (*n* = 3–13). All results are represented as means ± SEM; ****P < 0.0001 vs. mock control. Gray dots represent values from independent biological replicates. **d** Membrane localizations of GFP-tagged ABCG2 variants (green) are shown. Nuclear DNA was stained with DAPI (blue). Microscopy data are from duplicate experiments. Scale bars in microscopy images correspond to 20 µm. **e** Normalized mitoxantrone efflux activity of di-leucine valve mutants is plotted. Ko143-sensitive efflux activity is given as percentage activity of the WT control (*n* = 4–18). Bar graphs are shown as means ± SEM; ****P < 0.0001; **P < 0.01 vs. K86M. Gray dots represent values from independent biological replicates. **f** Vanadate-sensitive ATPase activities of ABCG2 variants stably expressed in HEK293 cells are quantified. Data are from independent experiments (*n* = 4–10), using three different batches of membrane preparations. Double mutation L554C L555C samples were prepared from four clones of stable cell line. Data are normalized and given as fold changes relative to WT. All data are means ± SEM; ****P < 0.0001; ***P < 0.001; **P < 0.01; *P < 0.1 vs. negative control. Gray dots represent values from independent biological replicates.

While the large central cavity is essential for substrate recognition or trapping (Supplementary Fig. 1c), the function of the upper cavity is most likely linked to the control of drug release. The top of the central cavity almost reaches the outer membrane bilayer leaflet, and is separated from the upper cavity by a valve-like constriction provided by residues G553 to T559, a short segment after TMH5 (Supplementary Fig. 1c). Interestingly, this short linker is highly conserved both in the mammalian ABCG family and in fungal PDR transporters (Supplementary Fig. 2c), indicating a critical functional role. The linker includes the di-leucine motif L554 and L555 in the middle, which sits on the top of the central cavity. L554 and L555 face each other in the half transporter interface in the core of ABCG2 dimer (Fig. 4a). Notably, residue F431, which is involved in Ko143 inhibitor binding as well as drug transport susceptibility^32,33^, resides just below L555 (Fig. 4a) and completes a constriction zone along the substrate translocation channel together with residue M549^19,20^. The position and proximity of L554 and L555 implies that they could form a valve-like gate essential for substrate translocation into the upper cavity. To verify this hypothesis, we generated ABCG2 single and double mutations of residues L554 and L555 by substituting with alanine, isoleucine, and cysteine, and functionally tested them in HEK293 cells (Fig. 4b–f). Strikingly, all L554 variants (L554A, L554I, and L554C) maintained almost normal ABCG2 surface levels, mitoxantrone efflux, and ATPase activities (Fig. 4b–f), demonstrating that L554 is not essential for ABCG2 biogenesis or function. By sharp contrast, reduction of the side chain size from the large hydrophobic leucine at position 555 to the smaller and less hydrophobic cysteine and alanine (L555C and L555A mutants) severely diminished protein levels due to reduced protein stability (Supplementary Fig. 3a), while maintaining the size of the side chain by introducing isoleucine (L555I), left mature protein levels, transport function, and ATP hydrolysis similar to WT ABCG2 (Fig. 4b–f). Of note, most leucine mutants showed normal membrane localization (Fig. 4d). Notably, the double mutants (L554A L555A and L554C L555C) showed significant defects on normalized mitoxantrone efflux, whereas L554I L555I mutant displayed decreased transport activity (Fig. 4e). Surprisingly, normalized ATPase activities were only moderately changed (Fig. 4f). The results demonstrate that ABCG2 requires at least one large hydrophobic residue at position 555 in the transporter core to allow for drug movement to the upper cavity but also for proper folding. The di-leucine motif is therefore critical for the efflux function, since it appears to form a hydrophobic seal by creating a valve. Finally, to confirm the close proximity of L554 and L555, we tested L554C and L555C mutant variants. Remarkably, immunoblotting of cells expressing the L554C and L555C variants under reducing conditions still revealed dimeric ABCG2 bands migrating above 130 kDa, with a stronger signal for L555C mutants. Hence, L555C must form a disulfide bond with L555C in the second ABCG2 half transporter. Notably, all mutants formed mainly dimers, migrating above 130 kDa under non-reducing conditions (Fig. 4b and Supplementary Fig. 3b). This proofs an ABCG2 dimer, in which the L554 and L555 residues are in juxtaposition across the dimer interface.

### Valve mutations disrupt the seal and enable water flow

**Fig. 5:**
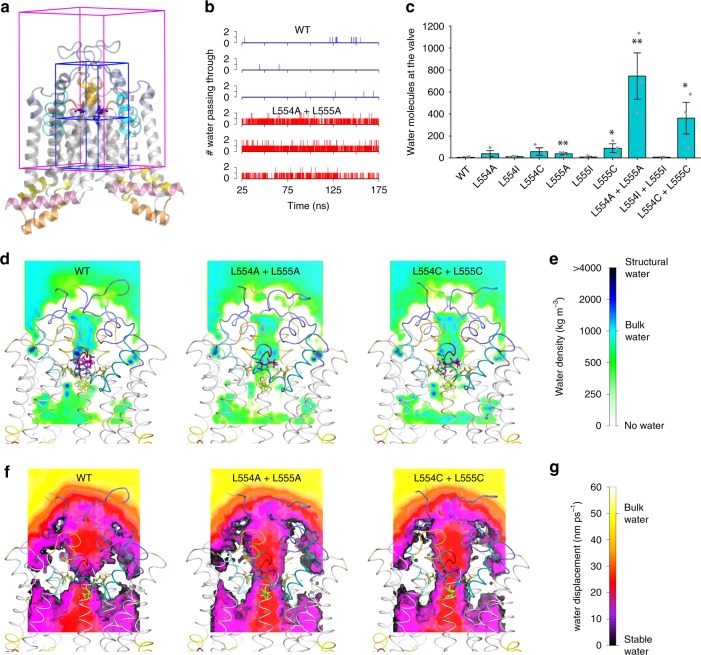
The di-leucine valve regulates water flow between central and upper cavities. **a** The spatial density of water was calculated in a 4 × 4 × 6 nm box surrounding the di-leucine valve (full magenta box). Blue boxes of total size 2 × 2 × 4 nm sides were used to determine water flow through the leucine valve by counting the number of water molecules exchanges between the cavities, analyzed in consecutive trajectory frames separated by 10 ps. Box boundary coincides with the di-leucine valve. **b** Water molecules passing through the di-leucine valve vs. time in WT and L554A L555A double mutant simulations. **c** Number of water molecules passing through the leucine valve averaged over three parallel runs using 150 ns for analysis. All data are shown as mean ± SEM. **P < 0.01 and *P < 0.1 vs. WT. Gray dots refer to values from independent experiment. **d** A slice of spatial water density shows water presence around the leucine valve and transport path in ABCG2. The map is the average of three parallel simulations using trajectories of 150 ns for the mutants and 500 ns for WT. **e** The color scale of the spatial water density is shown for **d**. **f** A slice of mean water displacement shows water dynamics around the leucine valve and transport path in ABCG2. The map is the average of three parallel simulations using trajectories of 150 ns for the mutants and 500 ns for WT. **g** The color scale for the mean water displacement is shown for **f**.

The di-leucine valve separates the upper and central cavities along a substrate translocation channel, which appears very narrow at the di-leucine valve (Fig. 4). However, none of the available ABCG2 structures suggest the presence of water molecules within the translocation channel. Thus, we performed 175-ns-long MD simulations to probe for the presence of water. To ensure equilibrated conformations and stable trajectories, we defined the initial 25 ns as equilibration time. Valve-disrupting mutants showed a strong local effect on water distribution, highlighted by substantial changes in spatial density surrounding the di-leucine valve (Fig. 5). The quantification of water exchange between the central and the upper cavity showed minor water movement through the valve for most constructs (Supplementary Fig. 5 and Fig. 5b, c). By sharp contrast, the double alanine L554A L555A mutant, as well as the double cysteine L554C L555C mutants, showed increased water movements when compared to the WT control (Supplementary Fig. 5 and Fig. 5b, c).

The kinetics of water movements showed a limited number of single or double water transitions (Fig. 5f). Of note, water movement between the central and the upper cavity appeared to be rare events, with single water transitions occurring at about every ~5 ns. However, the propensity of water movements dramatically increased in the double alanine L554A L555A and in the double cysteine L554C L555C mutants. The behavior of water molecules was further evaluated in terms of their spatial density (Fig. 5d, e and Supplementary Movies). The spatial density analysis highlights areas filled with water, and quantifies the propensity for water presence, indicating a marked difference between WT with the double alanine and double cysteine mutants. While the hydrophobic di-leucine valve prevents water flow between the central cavity and the upper cavity, the seal is weakened in the double mutants that contain less hydrophobic smaller residues, allowing for constitutive water flow through the substrate translocation path. The mutations change the function of the valve, thus allowing for water passing through the valve including substrate movement, which provides an explanation for altered mitoxantrone transport.

**Fig. 6:**
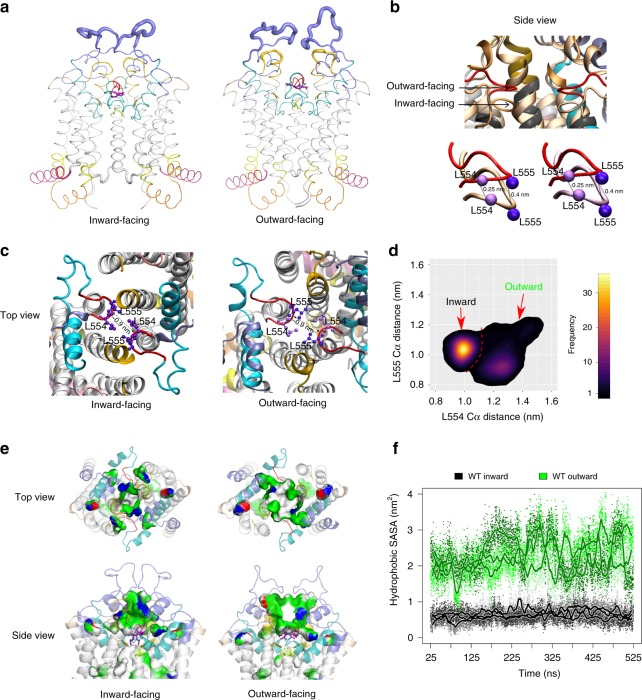
Conformational dynamics between inward- and outward-facing states. **a** MD simulations reveal conformational dynamics of the inward- and outward-facing states, quantified as root mean square fluctuations (RMSFs), and averaged over the three independent replicates. The results were highlighted in the structure by adapting the thickness of the respective backbone sections. The elbow helix (pink) followed by TMH1–6 helices (gray), cytoplasmic distal TMH2, and the TMH3 helical part (yellow), ECL1 (golden), ECL2 (old rose), the short loop after TMH5 (red), the re-entry helix (cyan), and the large extracellular ECL3 (violet). **b** The zoom-in side views show the overlay of the di-leucine valve between inward- (pale orange) and outward-facing (red) states. The Cα atoms of leucine 554 and 555 are in pale and dark purple balls, respectively. **c** The top view at the di-leucine valve of both states. Dotted lines indicate the distance in nm. **d** Di-leucine valve distance probability plots, averaged over the three independent simulations of outward and inward-facing ABCG2. **e** Expansion of the upper cavity. Slice through the upper cavity of the inward-facing and the outward-facing structures showing the polarity of cavity-facing color-coded residues; white (hydrophobic), green (polar), blue (positive charge), and red (negative charge). **f** Quantification of the solvent-exposed hydrophobic surface area (SASA) in the upper cavity. Data were obtained from three independent parallel MD simulations of 500 ns for WT in the inward- and outward-open conformations, with 10 ps sampling rate (small dots) and smoothened with a running average of 1 ns (thick line).

Further, we performed MD simulations for up to 500 ns for each replicate in three independently assembled membrane-embedded WT ABCG2 structures to reveal the dynamics of the inward- to outward-facing switch, as well as conformational dynamics of the TMDs, the central cavity, the di-leucine valve, as well as the upper cavity and the polar roof (Fig. 6). Interestingly, conformational flexibility was largely confined to a short ELC3 stretch between the disulfide bond connected C592 and C608 residues (Supplementary Fig. 1e and Fig. 6a), which also holds the 5D3 binding epitope^34^. By contrast, the lateral part of ELC3, including the re-entry helix, ECL1 and ECL2 showed restricted flexibility comparable to the TMHs or the intracellular elbow helix. These data strongly suggest that 5D3 Fab fragments can restrain ECL3 dynamics. Although 5D3 had only minor effects on overall ABCG2 structure, it seemed to impair the transport function as previously noted^14^. Nonetheless, these data confirm the existence of an upper cavity, and also demonstrate that ECL3 forming part of the upper lid is dynamic and thus accessible from the outside. The results also indicate a vertical upward shift of the di-leucine valve of ~0.25–0.4 nm in the outward-facing state, yet maintaining the hydrophobic seal that separates the central cavity from the upper cavity (Fig. 6b, c). Although the distance between the backbones from each monomer at the di-leucine valve remained at around 0.9 nm in both states, the dynamics in this core region was different (Fig. 6d). The distance distribution profiles revealed that the di-leucine valve is rigid in the inward-facing conformation, whereas it becomes more flexible in the outward-facing conformation. Notably, the geometry of the di-leucine valve differs between the two states. In the inward-facing state, residue L554 and L555 showed a single, well-defined geometry with similar Cα atom distances of 1.0–1.1 nm. However, in the outward-facing state, the prevalent distance between the Cα atoms of L554 increased to 1.3 nm, while the Cα atoms of L555 distance were closer (0.9 nm). Interestingly, the di-leucine valve was more flexible and did not show a single conformation, since distances ranged from 1.1 to 1.5 nm for L554 and 0.8 to 1.3 nm for L555. These local structural changes may mainly represent a consequence of global structural changes in the TMDs. There is minimal conformational overlap between two states, showing that the di-leucine valve has some flexibility and can adopts distinct conformations, which could enable the outward-facing state to open in the presence of substrate.

Finally, the upper cavity is surrounded by the distal regions of the TMHs and by all extracellular ECLs, including the re-entry helix. The MD data indicated that both volume and hydrophobic surface of the upper cavity is much smaller in the inward-facing state. Thus, cavity size dramatically expanded to a bowl shape during the switch to the outward-facing conformation, while the upper cavity still remaining semi-sealed. Notably, the outward-facing state held an at least twice as large hydrophobic surface area in upper cavity core (Fig. 6e, f), which was open to the extracellular space, perhaps preceding drug release.

## Discussion

We have recently dissected the molecular mechanism of the intracellular transmission interface during the transport cycle of ABCG2^18^. However, the role of the extracellular roof and how drug transport is accomplished or regulated through a putative central translocation channel has remained ill-posed. Here, we take advantage of available ABCG2 cryo-EM structures^14,19^, including an improved structure that appeared during the preparation of this work^19,20^ to explore the role of the extracellular lid-like roof and the di-leucine valve in the transporter core as key control elements for the catalytic cycle.

The structural arrangement of ABCG2-MZ29-Fab (PDB ID: 6ETI) in the inward-facing state illustrates a unique fold, which is similar to the X-ray structure of the human ABCG5/G8 heterodimer^13^. The core of drug-free ABCG2 appears to hold two cavities present in both the ATP-free state and in the holo-state. A large droplet-shaped central drug recognition/binding cavity in the transmembrane core ensures substrate binding or trapping, with several essential residues lining the inner cavity^17,18,35^. The small cavity in the extracellular region is separated from the central cavity by a short linker element present after TMH5 (Supplementary Fig. 1c). This stretch carries a di-leucine motif in the middle that seems to function as a molecular valve separating the central and upper cavities, which is subtending the extracellular polar roof (Supplementary Fig. 1c). Moreover, the roof architecture covers the upper cavity and acts as a restricted diffusion barrier. Indeed, MD simulations hint a rather stable R426 E585 salt bridge (Fig. 3g). It is therefore tempting to speculate that the roof controls both substrate release and prevents prolonged substrate residence in the upper cavity, perhaps to avoid substrate re-binding or ABCG2 inhibition.

In addition, ECL3 is stabilized by an intra-molecular disulfide bond between C592 and C608, as well as the intermolecular disulfide bond between C603 from both ABCG2 monomers^30,31^. Interestingly, at least one charged residue in each extracellular element is essential for function and proper targeting of ABCG2 (Figs. 1 and 2), demonstrating the crucial role of the lid-like roof structure. Importantly, residues R575 and E585 located in the putative re-entry helix, appear as most critical residues, since even conservative changes such as in E585D abrogate ABCG2 function, while others such as E611, E612, and D620 are pivotal for protein maturation (Figs. 1 and 2). Of note, D620 is a polymorphic site in human ABCG2, since D620N shows normal expression but moderately changed porphyrin and methotrexate transport^33,36^. These results suggest that conserved charged residues in the extracellular regions, except for E585, are required for proper protein folding, while conservative substitutions with identical-charge residues maintain ABCG2 function (Fig. 2).

Based on our data, we propose that human ABCG2 harbors two barriers, which could control and restrict substrate translocation to the extracellular space. First, a highly conserved gated molecular valve stretching from G553 to T559 after TMH5. Second, the compactness of the roof-like architecture may provide a lid-like function by creating a diffusion barrier that could also prevent re-binding of substrates and thus avoid jamming of the outer cavity (Supplementary Fig. 1c). Of note, the available cryo-EM structures may affect transport-competent roof structures, owing to the high-affinity binding of Fab fragments, which was necessary for purification and particle generation^14,19,20^. It seems plausible that Fab-fragment interactions could interfere with dynamics of the roof during the conformational switch. Our MD simulations support this notion, since the compact roof shows conformational flexibility, perhaps allowing for or facilitating diffusional drug release from the upper cavity (Fig. 6a).

Interestingly, residue F431, which determines both inhibitor specificity and binding^19^ is located just below the di-leucine valve facing into the substrate translocation path. Hence, F431 may subtend the hydrophobic leucine seal between the central cavity and the upper cavity, strongly supporting our notion about a transport mechanism that engages this di-leucine valve^32,33^. Remarkably, L554 is dispensable for ABCG2 function, whereas L555, which is reaching into the hydrophobic core, seems to have a pivotal role for ABCG2 function (Fig. 4). Noteworthy, the L555A and L555C variants show diminished ABCG2 expression, but retain mitoxantrone efflux and ATPase activity. Strikingly, double alanine and double cysteine valve mutations impair efflux activity, but fully retain membrane localization and ATPase hydrolysis (Fig. 4). This data imply that drug-stimulated ATPase activity may not be required for the conformational switch, and suggest that basal ATP hydrolysis is not necessarily coupled to substrate transport, as shown before for ABCG2^18^, as well as for fungal PDR transporters^10,37–39^. Interestingly enough, both single mutations L554C and L555C retain at least partial transport function, but form covalent cross-links reaching across the dimer interface. This finding confirms the close proximity of the leucine residues, implying a narrow substrate translocation pathway. Nonetheless, the hydrophobic side chain of leucine at position 555 is necessary for function. Remarkably, double mutations to alanine and cysteine fully abrogated ABCG2 efflux, but retained both surface protein expression and ATPase activity (Fig. 4). MD simulations indicate that these mutants particularly impair the valve, allowing for increased water penetration (Fig. 5), whereas the cysteine mutants retaining mitoxantrone efflux also show sealing capabilities comparable to WT ABCG2.

We propose that a loss of a hydrophobic side chain may impede proper valve function through changes in geometry, size, flexibility, and hydrophobicity, as well as water flow, thereby affecting both substrate transport and rate of ATP consumption. This may explain the increased ATPase of the L555A mutant, or suggest that the valve of the L555A variant can no longer couple drug transport to ATP hydrolysis. Drug translocation requires at least one large aliphatic side chain and a leucine residue for a fully functional valve. Thus, the local microenvironment at the hydrophobic seal may strongly affect substrate movement, since most ABCG2 substrates are hydrophobic compounds^40–43^. Furthermore, we speculate that the sealing of the di-leucine valve could be triggered by ATP hydrolysis, as ATP consumption separates the NBDs and may thus provide a mechanical leverage to the valve via movements of the ICLs, the elbow helix and the TMDs. Interestingly, L555 may be part of the LxxL steroid-binding motif stretching from L555 to L558, the so-called cholesterol recognition amino acid consensus domain^44,45^. Indeed, the presence of ABCG2 in placental lipid raft domains and a possible transport regulation was recently reported^46^. However, it remains unclear at this point whether ABCG2 or any of the di-leucine variants show altered cholesterol dependency of drug transport. Reports on the functional role of sterol-binding site mutations in L554 and L558A were contradictory, perhaps due to differences in the lipid composition of the bacterial and insect cell membranes, which are also drastically distinct from mammalian cells^44,45^. Furthermore, residues L554 to L558 face the putative ABCG2 translocation channel, and are therefore unlikely to directly interact or sense cholesterol in the membrane as previously suggested^19^.

The ABCG2 structure with the bound inhibitor Ko143 derivative suggests that both F431 and L555 are part of an inhibitor-binding region^19^, which is fully consistent with phenotypes we observe for L555 variants. Moreover, F431 is a polymorphic site that potentially interacts with other ABCG2 inhibitors such as fumitremorgin C and sunitinib. Remarkably, the F431L mutation loses inhibitor susceptibility^32,33^. This is in striking similarity to the related yeast Pdr5 transporter, where a mutation of S1360 in TMH10 changes both substrate specificity and at the same time abolishes susceptibility to the FK506 inhibitor^11,47^. However, L555 cannot be the main residue for inhibitor binding, since our experimental data demonstrate that all mutations at L554 and L555 retain full and normal inhibition by Ko143 (Supplementary Fig. 4). Hence, we believe that the di-leucine valve is not part of the Ko143 inhibitor-binding site, but critical for substrate translocation towards the upper cavity. Of note, we cannot exclude that the Ko143 derivative MZ29, which was obtained by replacing the methyl side chain with the longer cyclo-pentyl moiety, can also interact with L555^19^, but further experiments will bring a clarification.

**Fig. 7:**
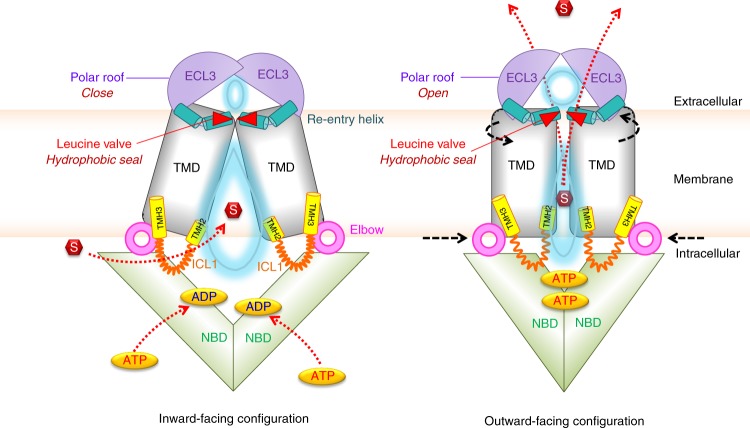
Model of ABCG2 transport cycle and drug movement through the di-leucine valve. The extracellular region, including the re-entry helix (cyan), di-leucine valve (red triangle), and ECL3 (violet), plays a vital role in translocation of substrate (red) from the central cavity (light blue water drop-shape) to the upper cavity (light blue oval) for drug release. Transmission interface, NBD (green), elbow helix (pink), distal parts of TMH2 and TMH3 (yellow), and ICL1 (orange) are essential for NBD-TMD interaction, which transmit the conformational switching, represented by black arrows. Red arrows indicate the directions of substrate or ATP translocations. See main text for further details.

ABCG2, although flexible in several segments, seems to display limited conformational dynamics due to a compact overall fold and because of essential intra- and inter-domain interactions^18^ that restrict overall mobility. For example, the extracellular architecture has at least two intra-molecular disulfide bonds connecting C592 and C608, and the intermolecular disulfide bond linking C603 in each monomer^29,30^, as well as a salt bridge between R426 of ECL1 and E585 of the re-entry helix. Indeed, our results demonstrate that the single mutants R426E or E585R completely abolish ABCG2 maturation, most likely owing to structural instability or due to a defective roof. Interestingly, the interactions at the transmission interface and the extracellular roof appear connected through the TMH1 and TMH2 helices. We thus speculate that TMH1 and TMH2 may provide a key mechanical link for the cross-talk that drives conformational switching during drug translocation (Supplementary Fig. 1a).

Although additional ABCG2 structures in the inward- and outward-facing drug-releasing states will be required at atomic resolution for a complete understanding of the transport cycle, our data are consistent with the following putative catalytic cycle (Fig. 7). In addition to drug trapping in the central cavity, which entails control by the transmission interface^18^, the drug transport cycle requires at least two key steps of regulation. First, the di-leucine valve controls substrate movement from the central cavity to the upper cavity. Importantly, the valve creates a tight hydrophobic seal at the top of central cavity that prevents water flow between both cavities in the inward-open state. In the outward-facing state, the valve changes conformation, thereby weakening the hydrophobic seal and enabling the peristaltic pressure to push substrates through the di-leucine valve. The pressure results from compression of the central cavity, which requires drug recognition, ATP binding, and NBD dimerization, the precise order of which remains unclear. Interestingly, the di-leucine valve maintains the overall gap size in the transporter core (Fig. 6c), which is large enough to allow for translocation of drugs with variable chemical spaces. Second, the polar roof of restricted flexibility forms a lid-like gate to control drug release from the upper cavity following a peristaltic motion originating from the inward-open state.

Noteworthy, both shape and volume of the central cavity is mainly determined by the arrangement of TMH1, TMH2, and TMH5 that surround the putative translocation channel. Additional TMHs around the central cavity show high flexibility, whereas the TMHs closer to the outer membrane interface are restricted in movement (Supplementary Fig. 6). Of course, it is tempting to speculate that intracellular drug trapping in the region of E446 or E451^18^ may also trigger rotational changes engaging the corresponding TMHs in each ABCG2 monomer into a cork-screw movement, which could contribute to or facilitate substrate translocation. This scenario is entirely compatible with the proposed squeezing mechanism as proposed here. In fact, rotational movements of TMHs could even facilitate the built-up of peristaltic pressure, since helix packing of TMHs would be different. Interestingly, both TMDs show rigid body motions, rotating around an axis parallel to the membrane plane through the center of TMDs, just below the di-leucine valve (Supplementary Fig. 6). Therefore, the amplitude of motions is largest at the cytosolic apex, smallest at the di-leucine valve, but increasing towards the extracellular roof. The re-entry helix in turn stabilizes the roof at the outer membrane interface by forming at least one verified salt bridge interaction with ECL1. The peristaltic pressure then expands the upper cavity and ECL3 acts as a critical lid component that concludes the ABCG2 cycle by opening the extracellular gate for substrate release. ATP hydrolysis occurs after drug release and aims to reset ABCG2 into the NBD-separated substrate trapping mode. The inward-facing state exposes both ATP-binding sites and sites for substrate trapping at residues E446 and E451 in the central cavity between the TMDs^18^. ATP-binding induces NBD dimerization and elicits the conformational switch involving the TMDs that drive translocation from the central cavity to the upper cavity.

Taken together, we provide compelling evidence that the human ABCG2 multidrug efflux transporter operates like a peristaltic drug pump gated by a hydrophobic valve and a flexible lid. Finally, our data suggest that inhibition of essential residues in the ABCG2 roof or in the di-leucine valve could hold promises for therapeutic intervention in case of drug-resistant neoplastic diseases, where ABCG2 has been notoriously known for playing detrimental roles.

## Methods

### Plasmids constructions

All mutations were made in vectors pcDNA3.1(−)-hABCG2 or pEGFPC1-hABCG2 plasmids as templates. Mutations were created by site-directed mutagenesis using Phusion DNA polymerase (NEB), followed by *Dpn*I digestion to eliminate DNA template before transforming into *Escherichia coli* (DH5α) for plasmid preparation. All plasmids constructs were confirmed by DNA sequencing. The primers in this study are indicated in the Supplementary Table 1.

### Mammalian cell culture and stable cell lines

Human embryonic kidney cells (HEK293) were cultivated from our frozen stocks at 37 °C, 5% CO_2_ with humidity in Dulbecco’s modified Eagle’s medium (DMEM) (Life Technologies, Rockville, MD, USA), supplemented with 10% (v/v) fetal bovine serum (FBS). Cells were seeded and maintained in a 6-well plate 1 day prior to transfection using self-made poly-ethyleneimine (PEI, Polysciences Inc., Germany) with 2 µg of plasmids as previously described^48^. At 48 h after transfection, cells were subjected for further experiments. The HEK293 stable cell lines were generated as described^49^. In brief, after 2 days post transfection, cells were seeded at low density between 50 and 500 cells into 10-cm dish containing culture medium supplemented with 0.9 g/l of G418. The culture medium was changed twice per week until the single colonies were observed. The single colony was isolated using cloning cylinder with trypsin digestion and transferred into 24-well plate prior to screening.

### Immunodetection

Cells were collected after trypsin digestion, followed by a washing step with ice-cold phosphate-buffered saline (PBS) (pH 7.4). Cell pellets containing 5 × 10^5^ cells were then lysed in ice-cold lysis buffer containing 50 mM Tris (pH 8.0), 120 mM NaCl, 1 mM EDTA, 2% Triton X-100, and freshly added protease inhibitor cocktail (Halt Protease and Phosphatase inhibitor Cocktail, Thermo Scientific). Cell debris was removed by centrifugation at 1200 × *g* for 2 min at 4 °C. The supernatants were collected and mixed with Laemmli sample buffer in the presence or absence of 100 mM dithiothreitol (DTT) before being subjected to sodium dodecyl sulfate (SDS)-polyacrylamide gel electrophoresis. Western blot analysis was performed according to standard protocols. After electroblotting onto nitrocellulose (GE Healthcare Life sciences, Freiburg, Germany), membranes were blocked by blocking solution and 5% bovine serum albumin in TBST buffer (Tris-buffered saline containing 0.1% Tween-20) at room temperature for 1 h. The primary mouse anti-ABCG2 antibody (BXP-21) (Santa Cruz Biotechnology, CA, USA) or rabbit anti-β-actin (D6A8) (Cell Signaling, MA, USA) were added at dilutions of 1:500 and 1:1000, respectively. The nitrocellulose membranes were washed three times with TBST for 15 min. Then, the IRDye^®^ 800CW secondary antibodies (LI-COR Biosciences, Homburg, Germany) against mouse (for anti-ABCG2) or rabbit (for anti-β-actin) (at dilution 1:10,000 in TBST) were used for further incubation at room temperature for 1 h. The signals were analyzed with the 800 channel of the Odyssey Imaging Systems and quantified by using Image Studio software version 2.1 (LI-COR^®^ Biosciences, Homburg, Germany). The bands of ABCG2 and β-actin were selected for quantification, and ABCG2 levels were normalized to β-actin before comparing with the expression level of WT ABCG2, which was set to 100%. Data were analyzed from at least three independent experiments.

### Functional assay

Transport activity of ABCG2 variants in HEK293 cells were measured using flow cytometry with the fluorescent compound mitoxantrone (Sigma-Aldrich, St. Louis, MO, USA), as a substrate, in the presence or absence of 1 µM Ko143 (Sigma-Aldrich, St. Louis, MO, USA) as ABCG2 inhibitor. In brief, cells were trypsinized and harvested in DMEM (containing 10% FBS) before washing with ice-cold PBS. The cell pellet containing 10^5^ cells per reaction were re-suspended in HPMI buffer (10 mM HEPES, 120 mM NaCl, 5 mM KCl, 400 µM MgCl_2_, 40 µM CaCl_2_, 10 mM NaHCO_3_, 10 mM glucose, 5 mM Na_2_HPO_4_, pH 7.4). The cells were pre-incubated with Ko143 for 5 min at 37 °C before adding mitoxantrone to a final concentration of 7 µM and further incubated for 20 min at 37 °C. The reactions were stop by placing the tube into ice-cold water for 5 min, followed by washing cells with ice-cold PBS and centrifuged at 600 × *g* for 2 min at 4 °C. The supernatant was removed and the cell pellets were re-suspended in ice-cold PBS for analysis using the BD FACSCalibur (Becton Dickinson, CA, USA). Mitoxantrone was determined with FL3 at excitation and emission wavelength at 488 and 670 nm, respectively. The viable cell population was selected and 10^4^ cells were collected for each data point. Data were analyzed using the FlowJo Software Inc. (Stanford University). The fluorescence intensity was corrected from unstained cells as a background. Signals were normalized to the activity in the presence of Ko143 inhibition, which was set to 100% inhibition. The results were represented as percentage of activity relative to WT ABCG2.

### Membrane protein preparation

HEK293 cells expressing ABCG2 variants were cultured in 10 cm dishes before washing with ice-cold PBS twice prior to harvesting. Cells pellets were re-suspended in ice-cold TMEP buffer (50 mM Tris, pH 7.0, 50 mM mannitol, 2 mM EGTA, and protease inhibitor cocktail), and lysed by passing the suspension through a 27-gauge needle using a syringe for 20 times. Debris was removed by centrifugation at 500 × *g* for 10 min; mitochondria protein was sedimented by centrifugation at 1200 × *g* for 5 min. Supernatants were collected prior to ultracentrifugation at 100,000 × *g* for 60 min. All procedures were always performed at 4 °C. The membrane proteins were aliquoted at 2 mg/ml and stored at −80 °C. The protein concentration was measured by the Bradford assay.

### ATPase assays

The vanadate-sensitive ATP hydrolysis activity of ABCG2 was examined using a crude membrane protein fraction from HEK293 cells. In brief, 5 µg of membrane protein was pre-incubated in ATPase assay buffer (50 mM MOPS (3-(*N*-morpholino)-propane-sulfonic acid), 50 mM KCl, 0.5 mM EGTA, 5 mM NaN_3_, 2.5 mM DTT, 1 mM ouabain, pH 7.0) in the presence or absence of 100 µM Na_3_VO_4_ (sodium orthovanadate) at 37 °C for 10 min. The reaction was started by adding 4 mM of ATP/Mg^2+^ in a total volume of 50 µl and incubated at 37 °C for 30 min. The reactions were stop by adding 40 µl of 5% SDS. Then, 100 µl of color reagent containing 3.33% (v/v) H_2_SO_4_, 0.48% (w/v), ammonium molybdate, 0.006% (w/v), antimony potassium tartrate, 5.7% (v/v) acetic acid, and 0.24% (w/v) ascorbic acid (freshly prepared) was added and incubated at room temperature for 30 min. The signal of released inorganic phosphate was determined at the wavelength 750 nm using microplate reader (VICTOR Nivo, Perkin-Elmer, Turku, Finland). An SDS-treated sample was used as a background. The vanadate-sensitive ATPase activity was calculated by subtracting from vanadate-treated sample. Data are represented as relative to WT.

### Confocal microscopy

HEK293 cells were seeded onto cover glass in 24-well plates. After 2 days in culture, cells were washed with ice-cold PBS and subsequently fixed with 4% formaldehyde in PBS at room temperature for 10 min. Slides were washed three times with PBS. Nuclei were stained with 5 µg/ml DAPI (4′,6-diamidino-2-pheny-lindole) in PBS at room temperature for 10 min. After washing cells with cold PBS three times, cells were incubated in 100 mM glycine for 15 min at room temperature. The cover slips were washed again in PBS before mounting on glass slides with mounting medium. Slides were dried in the dark and protected from light overnight before imaging with a Zeiss LSM700 confocal microscope using ZEN 2012 as analysis software.

### Protein stability analysis

Stability of protein was determined by using the cycloheximide chase assay. Briefly, stable cell lines expressing ABCG2 mutants were seeded and cultured for overnight in 24-well plates with 1 ml of DMEM (10% FBS, 1.5 mg/ml G418). After removing the medium, cells were treated with cycloheximide (Sigma-Aldrich, St. Louis, MO, USA) at a final concentration of 100 µg/ml (containing 0.1% dimethyl sulfoxide) for 0, 1, 3, 6, 12, and 24 h. At the indicated time points, cells were collected by trypsin digestion and harvested by centrifugation at 15,000 × *g* for 1 min at 4 °C. Cell pellets were lysed in protein lysis buffer containing freshly added protease inhibitor cocktail before performing western blot analysis with mouse anti-ABCG2 (BXP-21) or rabbit anti-β-actin (D6A8) antibodies as per the procedure described above. Quantification used the Image Studio software version 2.1 (LI-COR^®^ Biosciences, Homburg, Germany). Regions of measurements for both ABCG2 and β-actin were selected.

### MD simulations

The inward-open cryo-EM structure of ABCG2 (PDB ID: 6ETI^19^) was selected for the MD simulation. The missing last residue S655 was added using Pymol^50^, the intra- and intermolecular disulfide bridges were kept intact, while the missing N-terminus, the long loop between the first and the second β-strand of the core domain of the NBD, and the loop connecting the NBD with the TMD were not modeled, because introduction of long loops without suitable template structures usually decrease the reliability of models. The truncation residues were neutralized (acetyl and methyl for N and C terminals, respectively) to prevent the introduction of additional charges. The membrane lipid environment was assembled and equilibrated before production runs by first inserting ABCG2 in a 70/30 mol% POPC/cholesterol membrane^51^ containing membrane in coarse grain (CG) representation using the MARTINI force field^52,53^, surrounded with water and 150 mM NaCl using the “insane” script^54^, resulting in a simulation box of 12 × 12 × 13 nm. Three independently assembled CG systems were energy-minimized and simulated for 1 µs to equilibrate the membrane environment, while restraining the protein conformation.

All simulations used GROMACS version 2018.1^55^. A complete list of simulation parameters is given in Supplementary Table 2. All equilibrated CG systems were converted to an all-atom representation^56^. The initial ABCG2 structure replaced the back-mapped transporter to exclude spurious structural distortions introduced by the conversion procedure and inserted into the membrane using the *membed* procedure^57^ to relax possible local atom overlaps. MgATP was thereby included by placement into the nucleotide-binding sites of NBDs as observed in other ABC transporters^58^. Protein and solvent were described using the amber99sb-ildn force field^59^, POPC, and cholesterol by Slipid^60,61^ and ATP as described elsewhere^58^. The point mutations of the di-leucine valve (L554A, L554I, L554C, L555A, L555I, L555C, and the double mutations L554 L555A, L554 L555I, L554 L555C) were subsequently introduced. The final assembled systems were energy minimized and equilibrated in four steps of 2.5 ns each by slowly releasing the position restrains (1000, 100, 10, and 1 kJ/mol/nm) active on the Cα atoms of ABCG2 and on MgATP. The production runs of unrestrained systems were carried for 175 ns for mutants and 525 for WT; the first 25 ns of the production runs were excluded from analysis as equilibration stage; each system was independently simulated three times. See Supplementary Table 2 for simulation parameters for the production runs.

### Data and statistical analysis

All values in this study are represented as mean + SEM, unless stated otherwise. All in vitro experiments were performed in at least three independent assays. Figures and statistical analyses were generated by using R studio and GraphPad Software Inc. (San Diego, CA, USA) Prism version 5.00.

In silico data were analyzed by the GROMACS package, R and Python scripts using the MD Analysis package, v0.19.2^62,63^. For visualization, VMD^64^ v.1.9.3 and Pymol v.1.8.4 were used.

### Reporting summary

Further information on research design is available in the Nature Research Reporting Summary linked to this article.

## Supplementary information

Supplementary Information

Supplementary Movie 1

Supplementary Movie 2

Supplementary Movie 3

Supplementary Movie 4

Supplementary Movie 5

Supplementary Movie 6

Supplementary Movie 7

Supplementary Movie 8

Supplementary Movie 9

Supplementary Movie 10

Reporting Summary

Description of Additional Supplementary Files

## Data Availability

Data supporting the findings of this manuscript are available from the corresponding author upon reasonable request. A reporting summary for this Article is available as a Supplementary Information file. The source data underlying Figs. 1c, d, 2b, c, 3c–e, g–i, 4c, e, f, and 5c are separately provided as a Source Data file.

## Source data

Source Data
