# Supplementary material for: The ABCG2 multidrug transporter is a pump gated by a valve and an extracellular lid
Source: Nat Commun. 2019 Nov 28;10:5433. doi: 10.1038/s41467-019-13302-2 (PMC6883074; doi:10.1038/s41467-019-13302-2)
Supplement: Supplementary file 1 — Supplementary Information [file 41467_2019_13302_MOESM1_ESM.pdf]

Supplementary Information

The ABCG2 Multidrug Transporter is a Pump Gated by a Valve and an Extracellular Lid

Narakorn Khunweeraphong[1], Daniel Szöllősi[2], Thomas Stockner[2] & Karl Kuchler[1,*]

From the

[1] Medical University of Vienna, Center for Medical Biochemistry, Max Perutz Labs Vienna, Campus Vienna Biocenter, Dr. Bohr-Gasse 9/2, A-1030 Vienna, Austria

&

[2]Medical University of Vienna, Center for Physiology and Pharmacology, Institute of Pharmacology, Währingerstrasse 13A, A-1090 Vienna, Austria

*** To whom corresponding may be addressed:**

Karl Kuchler, Medical University of Vienna, Center for Medical Biochemistry

Max Perutz Labs Vienna, Campus Vienna Biocenter, 1030 Vienna, Austria

e-mail: karl.kuchler@meduniwien.ac.at

Phone: +43-1-4277-61807; FAX: +43-1-4277-9618

Keywords: multidrug transporter, anticancer resistance, ABCG2 structure, extracellular membrane interface, leucine valve, catalytic cycle

Supplementary figures and legends

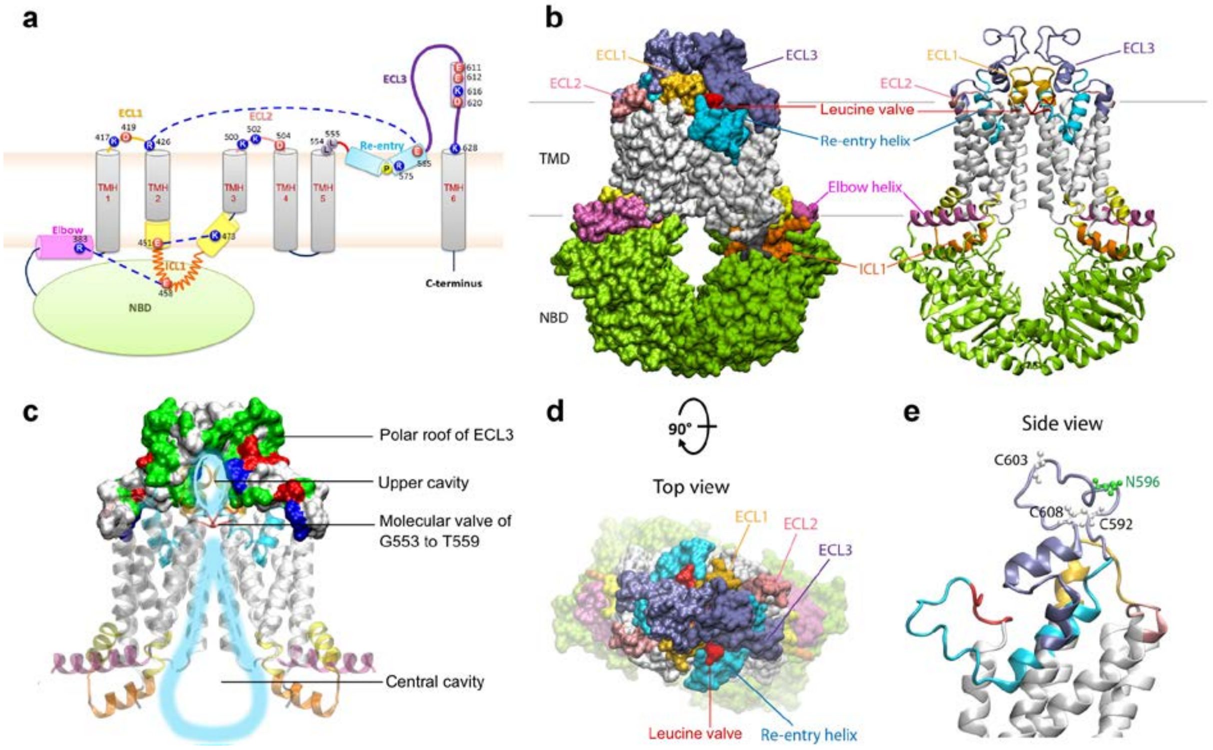

Supplementary Figure 1. Architecture of the extracellular domains of ABCG2.
(**a**) The structure of ABCG2 shows the N-terminal nucleotide binding domain NBD (green) connected to the elbow helix (pink) followed by transmembrane helix TMH 1-6 (grey), the first intracellular loop ICL1 (orange), cytoplasmic distal TMH2, TMH3 helical part (yellow). The extracellular elements are ECL1 (golden), ECL2 (old rose), the short loop after TMH5 (red), the re-entry helix (cyan) and the large ECL3 (violet). Amino acid residues are shown as colored balls, positive (blue), negative (red), leucine (violet) and proline (yellow). The salt bridge interactions are indicated by blue dotted lines. (**b**) The cryo-EM structure of the homo-dimeric human ABCG2 transporter (ABCG2-MZ29-Fab (PDB ID: 6ETI)) is shown in space-filling mode (left) and ribbon representation (right). In the ATP-free state, the inward-open conformation shows a compact extracellular region, colored as in panel a. (**c**) The transmembrane domains (TMDs) of ABCG2 form a large water drop-like central cavity (blue), separated from an upper cavity (blue oval) by a molecular valve structure ranging from residue G553 to T559, a part of the short linker after TMH5 (red ribbon). ECL3, located on the top of the upper cavity depicted in polarity surface mode; polar residues (green), negatively charged residues (red), positively charged residues (blue), non-polar residues (white). (**d**) Top view of the outer ABCG2 architecture highlighting the polar roof. (**e**) Zoom-in membrane-side view rotated by 90° of ABCG2 in ribbon mode to show the extracellular membrane regions; positions of three cysteine residues (white balls-and-sticks) and asparagine (green balls-and-sticks) in ECL3 are given. A kinked re-entry helix (cyan ribbon) submerges into the outer leaflet, reaching into the membrane lipid bilayer.

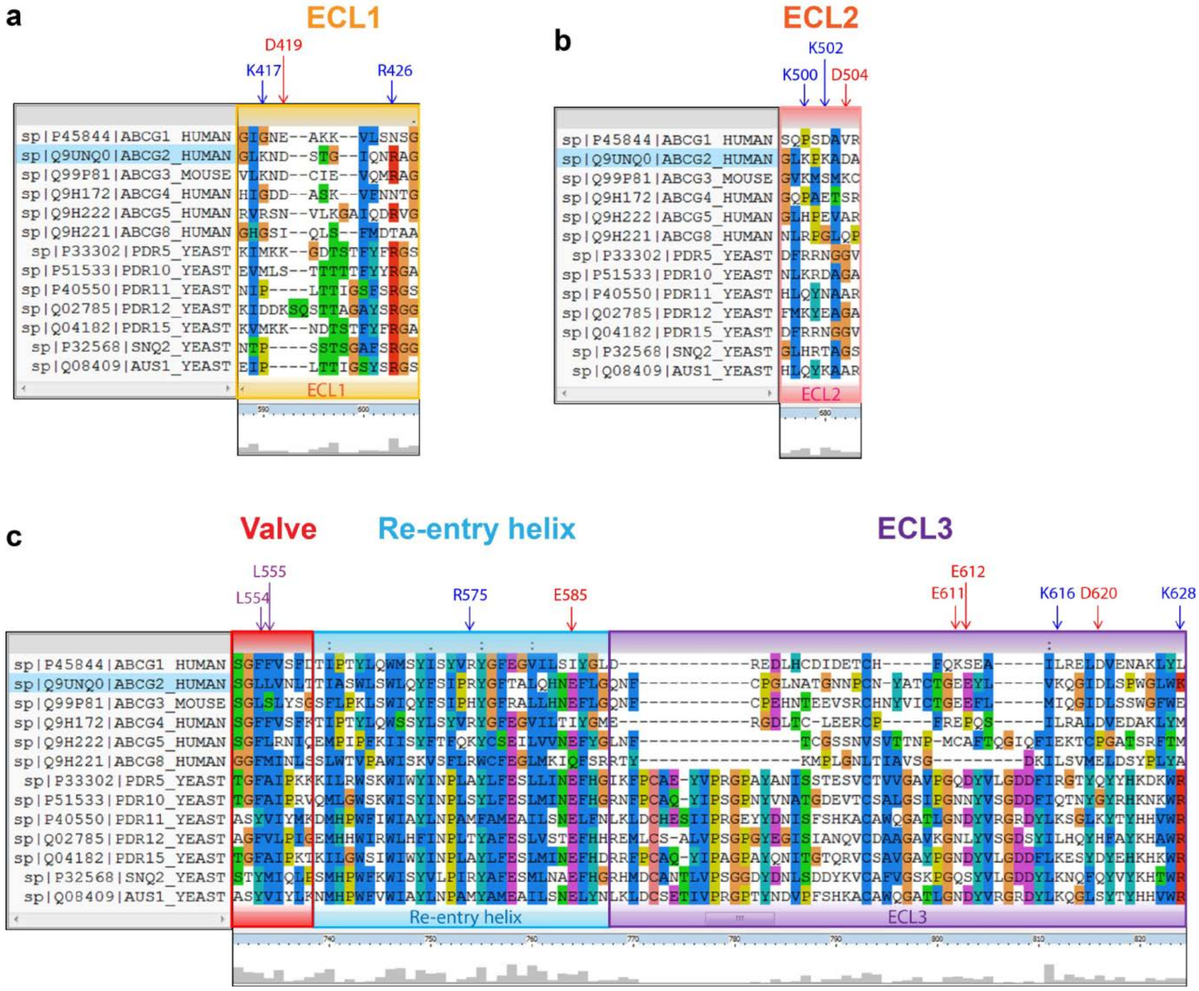

Supplementary Figure 2. Sequence alignments of mammalian ABCGs and yeast PDRs. The alignments were conducted using clustalX2. The conserved residues are highlighted and the conservation score shown at the bottom, indicated by the height of grey bars. The extracellular regions are marked by boxes: (**a**) ECL1 (golden), (**b**) ECL2 (old rose) and (**c**) short link after TMH5 (red), re-entry helix (cyan) and ECL3 (violet), respectively.

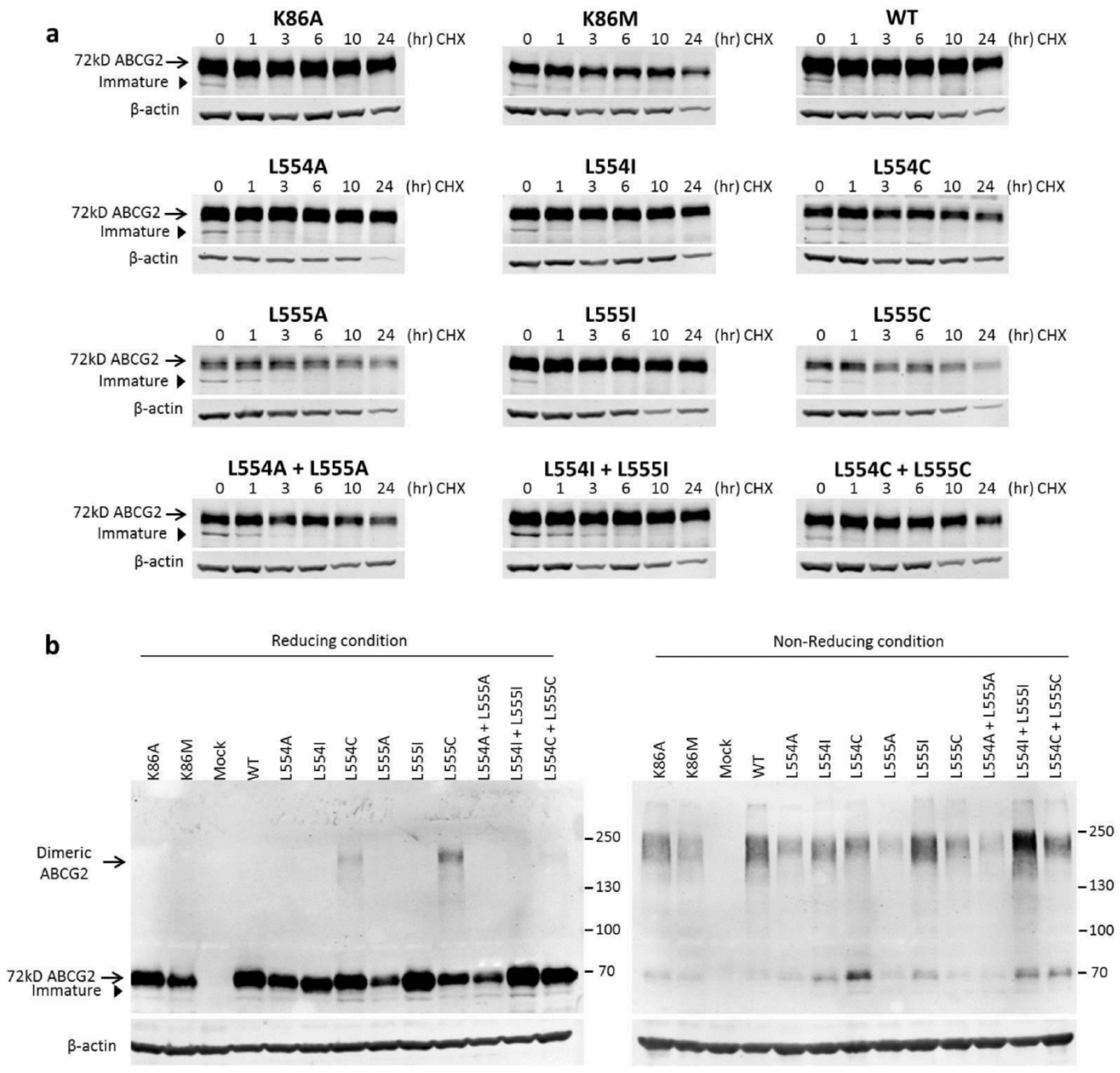

Supplementary Figure 3. Immunodetection of ABCG2 mutants in stable HEK293 cells.
(**a**) Proteolytic stability of ABCG2 mutants were analyzed in stable HEK293 cells. Cycloheximide was treated at indicated time point. Cells were lysed and subjected to immunoblotting using the monoclonal mouse anti-ABCG2 (BXP-21) antibody. The arrows indicate mature glycosylated protein at approximately 72 kDa, while arrow heads point to the immature bands. β-actin was used as an internal loading control. (b) Immunodetection of ABCG2 variants, stably expressed in HEK293 cells in reducing and non-reducing conditions and detected by the anti-ABCG2 (BXP-21) antibody. ABCG2 monomers migrate at approximately 72 kDa, while dimeric ABCG2 is located above. β-actin was used as an internal loading control.

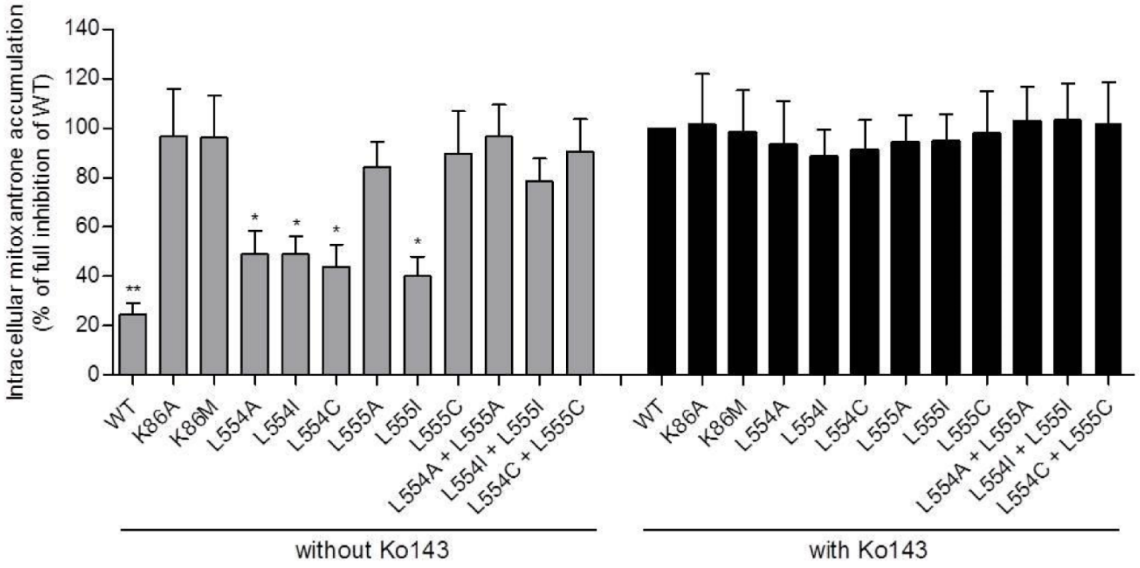

Supplementary Figure 4. Intracellular mitoxantrone accumulation in stable HEK293 cells. Mitoxantrone accumulation was quantified in the presence (black bars) and absence (grey bars) of Ko143 after incubation for 20 min at 37 °C. Mitoxantrone accumulation is given as percentage full inhibition relative to WT. Data are from several independent experiments (n = 7) and represented as means with ± SEM; **$P < 0.01$; *$P < 0.1$ vs. K86M as negative control.

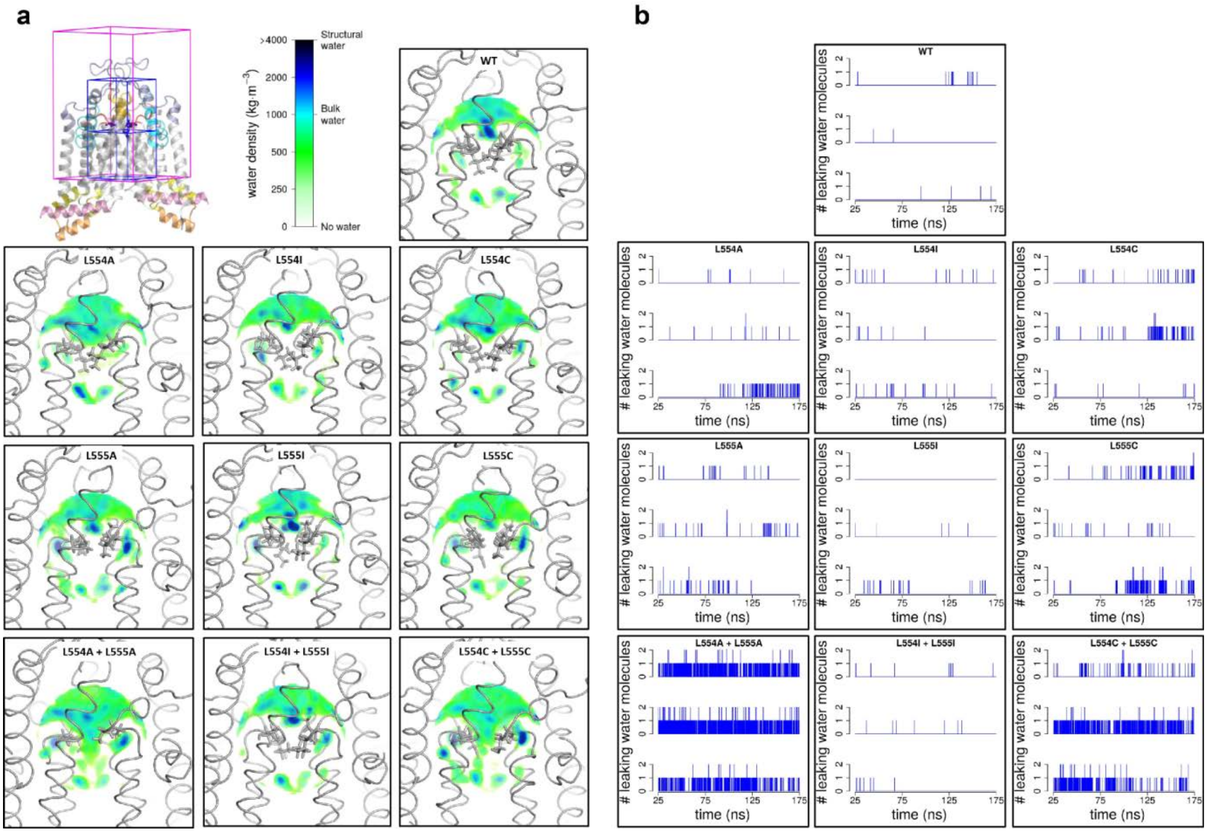

Supplementary Figure 5. Water movement through the di-leucine valve.

(**a**) Spatial density of water was calculated in a 4x4x6 nm box centered at the di-leucine valve (magenta box). Two blue boxes of 2x2x2 nm sides were used to determine water flow through the di-leucine valve by counting the number of exchanged water molecules in consecutive simulation frames of 10 ps separation. (**b**) Waters passing through the di-leucine valve were counted and plotted versus time. The counting was based on two selection regions (see blue boxes) and a value was picked if a water molecule left one selection and entered another at the same time.

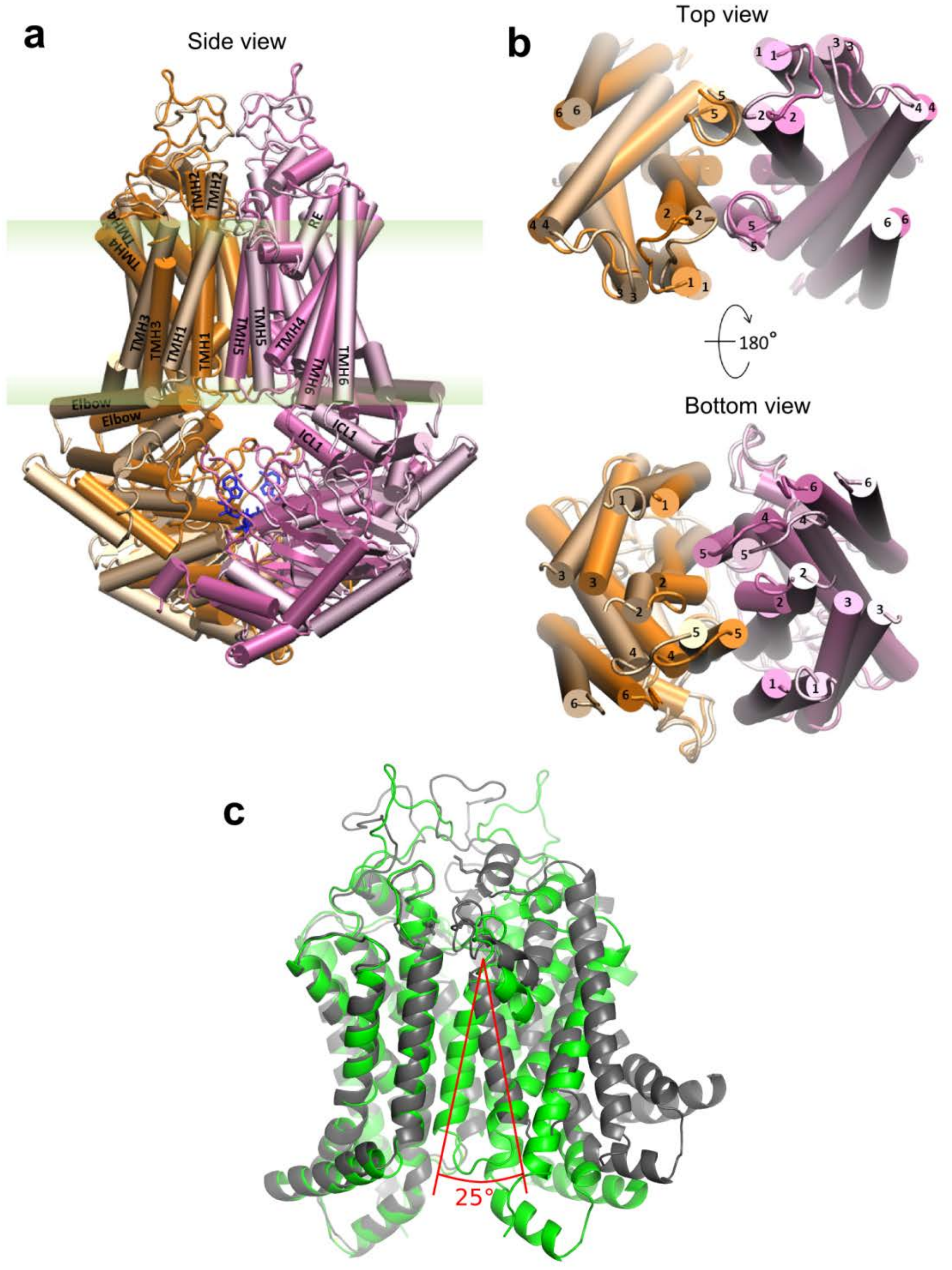

Supplementary Fig. 6: Conformational changes between inward- and outward-facing states.

(**a**) Overlay of dimeric ABCG2 between inward-facing (pale orange and pale pink) and outward-facing (dark orange and dark pink) states (PDB ID: 6ETI and 6HBU, respectively). (**b**) Top-view and bottom-view at both membrane leaflets of the TMD indicate movements of six TMHs at the membrane border during the catalytic cycle (NBD- and extracellular region-removed for clarity). (**c**) Conformational changes of the TMDs during switch between the inward- (grey) and the outward-facing states (green). The ABCG2 dimer was fitted onto TMD1. The movement of TMH5 that stretch along the central cavity was measured in both states and highlighted as the angle in red line.

Supplementary Table 1: Oligonucleotide primers used to generate ABCG2 mutations.

Mutants	Forward primer (5'->3')	Reverse primer (5'->3')
K417E	CTTTGGGCTAgaaAATGATTCTACTGG	CCAGTAGAATCATTttcTAGCCCAAAG
D419K	GGGCTAAAAAATaAaTCTACTGGAATCC	GGATTCCAGTAGAtTtATTTTTTAGCCC
D419E	GCTAAAAAATGAaTCTACTGGAATC	GATTCCAGTAGAtTCATTTTTTAGC
R426E	GAATCCAGAACgaaGCTGGGGTTCTC	GAGAACCCCAGCttcGTTCTGGATTC
R426K	GAATCCAGAACaaaGCTGGGGTTCTC	GAGAACCCCAGCtttGTTCTGGATTC
K500E	GTTAGGATTGgagCCAAAGGCAGATGC	GCATCTGCCTTTGGctcCAATCCTAAC
K500H	GTTAGGATTGcacCCAAAGGCAGATGC	GCATCTGCCTTTGGgtgCAATCCTAAC
K500R	GTTAGGATTGaggCCAAAGGCAGATGC	GCATCTGCCTTTGGcctCAATCCTAAC
K500M	GTTAGGATTGatgCCAAAGGCAGATGC	GCATCTGCCTTTGGcatCAATCCTAAC
K500A	GTTAGGATTGgcgCCAAAGGCAGATGC	GCATCTGCCTTTGGcgcCAATCCTAAC
K502E	GATTGAAGCCAgagGCAGATGCCTTC	GAAGGCATCTGCctcTGGCTTCAATC
D504K	GCCAAAGGCAaAaGCCTTCTTCGTTATG	CATAACGAAGAAGGCtTtTGCCTTTGGC
D504E	CCAAAGGCAGAaGCCTTCTTCG	CGAAGAAGGCtTCTGCCTTTGG
R575E	TCAGCATTCCAgaaTATGGATTTACGG	CCGTAAATCCATAttcTGGAATGCTGA
R575D	TCAGCATTCCAgacTATGGATTTACGG	CCGTAAATCCATAgtcTGGAATGCTGA
R575K	TCAGCATTCCAaaaTATGGATTTACGG	CCGTAAATCCATAtttTGGAATGCTGA
E585K	GCAGCATAATaAATTTTTGGGAC	GTCCCAAAAATTtATTATGCTGC
E585R	GCAGCATAATcgATTTTTGGGAC	GTCCCAAAAATcgATTATGCTGC
E585D	GCAGCATAATGAtTTTTTGGGAC	GTCCCAAAAAaTCATTATGCTGC
E611K	CCAAATATTCTTtGCCAGTACATG	GTACTGGCGAAaAATATTTGG
E611D	CATGTACTGGCGAtGAATATTTGG	CCAAATATTCaTCGCCAGTACATG
E612K	GTACTGGCGAAaAATATTTGG	CCAAATATTtTTCGCCAGTAC
E612D	GTACTGGCGAAGAtTATTTGG	CCAAATAaTCTTCGCCAGTAC
K616E	GAATATTTGGTAgagCAGGGCATCGATC	GATCGATGCCCTGctcTACCAAATATTC
D620K	GCAGGGCATCaAaCTCTCACCCTGG	CCAGGGTGAGAGtTtGATGCCCTGC
D620R	CAGGGCATCcgTCTCTCACCCTG	CAGGGTGAGAGAcgGATGCCCTG
D620E	CAGGGCATCGAaCTCTCACCCTG	CAGGGTGAGAGtTCGATGCCCTG
K628E	GGGGCTTGTGGgagAATCACGTGGCC	GGCCACGTGATTctcCCACAAGCCCC
L554A	GATTTTTTCAGGTgcGTTGGTCAATCTCAC	GTGAGATTGACCAACgcACCTGAAAAAATC
L554I	GATTTTTTCAGGTaTcTTGGTCAATCTCAC	GTGAGATTGACCAAgAtACCTGAAAAAATC
L554C	GATTTTTTCAGGTtgcTTGGTCAATCTCAC	GTGAGATTGACCAAgcaACCTGAAAAAATC
L555A	GATTTTTTCAGGTCTGgcGGTCAATCTCAC	GTGAGATTGACCgcCAGACCTGAAAAAATC
L555I	GATTTTTTCAGGTCTGaTcGTCAATCTCAC	GTGAGATTGACgAtCAGACCTGAAAAAATC
L555C	GATTTTTTCAGGTCTGTgcGTCAATCTCAC	GTGAGATTGACgcACAGACCTGAAAAAATC
L554A + L555A	GATGATTTTTTCAGGTgcGgcGGTCAATCTCACAACC	GGTTGTGAGATTGACCgcCgcACCTGAAAAAATCATC
L554I + L555I	GATGATTTTTTCAGGTaTcaTcGTCAATCTCACAACC	GGTTGTGAGATTGACgAtgAtACCTGAAAAAATCATC
L554C + L555C	GATGATTTTTTCAGGTtgcTgcGTCAATCTCACAACC	GGTTGTGAGATTGACgcAgcaACCTGAAAAAATCATC

Supplementary Table 2: Molecular dynamics simulation parameters

	coarse grain			all atom		
parameter	Energy minimization	equilibration	production	Energy minimization	equilibration	production
consecutive runs	1	1	1	50	4	1
length	100 ps	5 ns	1 µs	10 step	2.5 ns	50 ns
time step	0.01	0.02	0.02	0.002	0.002	0.002
cutoff scheme	Verlet	Verlet	Verlet	Verlet	Verlet	Verlet
nstlist	20	20	20	50	50	50
rlist	1.0	1.0	1.0	0.9	0.9	0.9
coulombtype	reaction-field	reaction-field	reaction-field	PME	PME	PME
rcoulomb	1.1	1.1	1.1	0.9	0.9	0.9
rvdw	1.1	1.1	1.1	0.9	0.9	0.9
tcoupl	-	v-rescale	v-rescale	-	v-rescale	v-rescale
temperature	-	310	310	-	310	310
tau-t	-	1.0	1.0	-	0.5	0.5
pcoupl	-	Berendsen	Parrinello-Rahman	-	Berendsen	Parrinello-Rahman
tau-p	-	12.0	12.1	-	5.1	20.1
lincs-iter	1	1	1	2	2	2
