# Supplementary material for: The ABCG2 multidrug transporter is a pump gated by a valve and an extracellular lid
Source: Nat Commun. 2019 Nov 28;10:5433. doi: 10.1038/s41467-019-13302-2 (PMC6883074; doi:10.1038/s41467-019-13302-2)
Supplement: Supplementary file 12 — Reporting Summary [file 41467_2019_13302_MOESM12_ESM.pdf]

nature research

Corresponding author(s): Karl Kuchler

Last updated by author(s): Aug 27, 2019

Reporting Summary

Nature Research wishes to improve the reproducibility of the work that we publish. This form provides structure for consistency and transparency in reporting. For further information on Nature Research policies, see Authors & Referees and the Editorial Policy Checklist.

Statistics

For all statistical analyses, confirm that the following items are present in the figure legend, table legend, main text, or Methods section.

n/a	Confirmed	
[ ]	[x]	The exact sample size (*n*) for each experimental group/condition, given as a discrete number and unit of measurement
[ ]	[x]	A statement on whether measurements were taken from distinct samples or whether the same sample was measured repeatedly
[ ]	[x]	The statistical test(s) used AND whether they are one- or two-sided<br>*Only common tests should be described solely by name; describe more complex techniques in the Methods section.*
[ ]	[x]	A description of all covariates tested
[ ]	[x]	A description of any assumptions or corrections, such as tests of normality and adjustment for multiple comparisons
[ ]	[x]	A full description of the statistical parameters including central tendency (e.g. means) or other basic estimates (e.g. regression coefficient) AND variation (e.g. standard deviation) or associated estimates of uncertainty (e.g. confidence intervals)
[ ]	[x]	For null hypothesis testing, the test statistic (e.g. *F*, *t*, *r*) with confidence intervals, effect sizes, degrees of freedom and *P* value noted<br>*Give P values as exact values whenever suitable.*
[x]	[ ]	For Bayesian analysis, information on the choice of priors and Markov chain Monte Carlo settings
[x]	[ ]	For hierarchical and complex designs, identification of the appropriate level for tests and full reporting of outcomes
[x]	[ ]	Estimates of effect sizes (e.g. Cohen's *d*, Pearson's *r*), indicating how they were calculated

Our web collection on statistics for biologists contains articles on many of the points above.

Software and code

Policy information about availability of computer code

Data collection	No software was used.
Data analysis	GraphPad Prism 5.00, R Studio, FlowJo7, Image Studio Ver 2.1, ZEN, VMD 1.9.2, ClustalX2, GROMACS, PYMOL, PYTHON

For manuscripts utilizing custom algorithms or software that are central to the research but not yet described in published literature, software must be made available to editors/reviewers. We strongly encourage code deposition in a community repository (e.g. GitHub). See the Nature Research guidelines for submitting code & software for further information.

Data

Policy information about availability of data

All manuscripts must include a data availability statement. This statement should provide the following information, where applicable:

- Accession codes, unique identifiers, or web links for publicly available datasets
- A list of figures that have associated raw data
- A description of any restrictions on data availability

Supplementary movies and source data for Figures 2, 3, 4, 5 and 6 are available online.

Field-specific reporting

Please select the one below that is the best fit for your research. If you are not sure, read the appropriate sections before making your selection.

[x] Life sciences [ ] Behavioural & social sciences [ ] Ecological, evolutionary & environmental sciences

For a reference copy of the document with all sections, see nature.com/documents/nr-reporting-summary-flat.pdf

Life sciences study design

All studies must disclose on these points even when the disclosure is negative.

Sample size	At least three biological independent replicates were performed in all experiments. Each single data point was represented as a grey dot in the graphs. Unpaired t-test was used for statistical analysis.
Data exclusions	No data excluded.
Replication	All replicates were successful.
Randomization	In this study, samples were not from animals or human. Randomization was not applicable.
Blinding	Blinding was not relevant in this study since samples from animal or human were not used. Blinding was not applicable.

Reporting for specific materials, systems and methods

We require information from authors about some types of materials, experimental systems and methods used in many studies. Here, indicate whether each material, system or method listed is relevant to your study. If you are not sure if a list item applies to your research, read the appropriate section before selecting a response.

Materials & experimental systems

n/a	Involved in the study	
[ ]	[x]	Antibodies
[ ]	[x]	Eukaryotic cell lines
[x]	[ ]	Palaeontology
[x]	[ ]	Animals and other organisms
[x]	[ ]	Human research participants
[x]	[ ]	Clinical data

Methods

n/a	Involved in the study	
[x]	[ ]	ChIP-seq
[ ]	[x]	Flow cytometry
[x]	[ ]	MRI-based neuroimaging

Antibodies

Antibodies used	Mouse anti-ABCG2 (BXP-21, Santa Cruz Biotechnology), rabbit anti-β-actin (D6A8, Cell Signaling), IRDye 800CW secondary antibodies against mouse or rabbit (LI-COR Biosciences)
Validation	All commercial antibodies were validated according to the companies. Positive and negative controls were included in every experiment.

Eukaryotic cell lines

Policy information about cell lines

Cell line source(s)	HEK293 (ATCC)
Authentication	No
Mycoplasma contamination	Cells were checked and no contamination from Mycoplasma.
Commonly misidentified lines<br>(See ICLAC register)	No

Flow Cytometry

Plots

Confirm that:

[ ] The axis labels state the marker and fluorochrome used (e.g. CD4-FITC).

[ ] The axis scales are clearly visible. Include numbers along axes only for bottom left plot of group (a 'group' is an analysis of identical markers).

[ ] All plots are contour plots with outliers or pseudocolor plots.

[x] A numerical value for number of cells or percentage (with statistics) is provided.

Methodology

Sample preparation	Samples were prepared from HEK293 cells by trypsinization. After incubation with substrate with/without inhibitor, cells were washed and resuspend in cold PBS for measurement. Details are provided in Materials and methods.
Instrument	FACSCalibur
Software	FlowJo 7
Cell population abundance	Cell populations were selected according to the size (forward-scatter) and complexity (side-scatter). Debris were excluded. 10,000 events per data point were collected for analysis.
Gating strategy	Cell populations according to the size (forward-scatter) and complexity (side-scatter) were gated for analysis. Debris were excluded. 10,000 events per data point were collected.

[ ] Tick this box to confirm that a figure exemplifying the gating strategy is provided in the Supplementary Information.
