# Supplementary material for: The ABCG2 multidrug transporter is a pump gated by a valve and an extracellular lid
Source: Nat Commun. 2019 Nov 28;10:5433. doi: 10.1038/s41467-019-13302-2 (PMC6883074; doi:10.1038/s41467-019-13302-2)
Supplement: Supplementary file 13 — Description of Additional Supplementary Files [file 41467_2019_13302_MOESM13_ESM.pdf]

Supplementary movie legends

Title: Supplementary Movie 1

The di-leucine valve regulates water flow between central and upper cavities of WT ABCG2.

Description: (**Upper left panel**) The zoom-in of WT ABCG2 at the di-leucine valve (red) shows the side chains of L554 (light magenta), L555 (dark magenta), F431 (dark green) and M549 (light green). (**Upper middle panel**) The spatial water density shows water around the di-leucine valve and transport part of ABCG2. The map is the average of 3 parallel simulations using trajectories of 500 ns. (**Upper right panel**) The color scale of the spatial water density is shown for the upper middle panel. (**Lower left panel**) The zoom-in of WT ABCG2 at di-leucine valve with the same labelling in the upper left panel shows the water molecule (light blue). (**Lower middle panel**) The mean water displacement shows water dynamics around the valve and the transport path in ABCG2. The map is the average of 3 parallel simulations using trajectories of 500 ns. (**Lower right panel**) The color scale for the mean water displacement is shown for the lower middle panel.

Title: Supplementary Movie 2

The di-leucine valve regulates water flow between central and upper cavities of L554A mutant.

Description: (**Upper left panel**) The zoom-in of L554A mutant at the valve (red) shows the side chains of L554A (light magenta), L555 (dark magenta), F431 (dark green) and M549 (light green). (**Upper middle panel**) The spatial water density shows water around the valve and transport part of ABCG2. The map is the average of 3 parallel simulations using trajectories of 150 ns. (**Upper right panel**) The color scale of the spatial water density is shown for the upper middle panel. (**Lower left panel**) The zoom-in of L554A mutant at the valve with the same labelling in the upper left panel shows the water molecule (light blue). (**Lower middle panel**) The mean water displacement shows water dynamics around the valve and the transport path in ABCG2. The map is the average of 3 parallel simulations using trajectories of 150 ns. (**Lower right panel**) The color scale for the mean water displacement is shown for the lower middle panel.

Title: Supplementary Movie 3

The di-leucine valve regulates water flow between central and upper cavities of L554I mutant.

Description: (**Upper left panel**) The zoom-in of L554I mutant at the valve (red) shows the side chains of L554I (light magenta), L555 (dark magenta), F431 (dark green) and M549 (light green). (**Upper middle panel**) The spatial water density shows water around the valve and transport part of ABCG2. The map is the average of 3 parallel simulations using trajectories of 150 ns. (**Upper right panel**) The color scale of the spatial water density is shown for the upper middle panel. (**Lower left panel**) The zoom-in of L554I mutant at the valve with the same labelling in the upper left panel shows the water molecule (light blue). (**Lower middle panel**) The mean water displacement shows water dynamics around the valve and the transport path in ABCG2. The map is the average of 3 parallel simulations using trajectories of 150 ns. (**Lower right panel**) The color scale for the mean water displacement is shown for the lower middle panel.

Title: Supplementary Movie 4

The di-leucine valve regulates water flow between central and upper cavities of L554C mutant.

Description: (**Upper left panel**) The zoom-in of L554C mutant at the valve (red) shows the side chains of L554C (light magenta), L555 (dark magenta), F431 (dark green) and M549 (light green). (**Upper middle panel**) The spatial water density shows water around the valve and transport part of ABCG2. The map is the average of 3 parallel simulations using trajectories of 150 ns. (**Upper right panel**) The color scale of the spatial water density is shown for the upper middle panel. (**Lower left panel**) The zoom-in of L554C mutant at the valve with the same labelling in the upper left panel shows the water molecule (light blue). (**Lower middle panel**) The mean water displacement shows water dynamics around the valve and the transport path in ABCG2. The map is the average of 3 parallel simulations using trajectories of 150 ns. (**Lower right panel**) The color scale for the mean water displacement is shown for the lower middle panel.

Title: Supplementary Movie 5

The di-leucine valve regulates water flow between central and upper cavities of L555A mutant.

Description: (**Upper left panel**) The zoom-in of L555A mutant at the valve (red) shows the side chains of L554 (light magenta), L555A (dark magenta), F431 (dark green) and M549 (light green). (**Upper middle panel**) The spatial water density shows water around the valve and transport part of ABCG2. The map is the average of 3 parallel simulations using trajectories of 150 ns. (**Upper right panel**) The color scale of the spatial water density is shown for the upper middle panel. (**Lower left panel**) The zoom-in of L555A mutant at the valve with the same labelling in the upper left panel shows the water molecule (light blue). (**Lower middle panel**) The mean water displacement shows water dynamics around the valve and the transport path in ABCG2. The map is the average of 3 parallel simulations using trajectories of 150 ns. (**Lower right panel**) The color scale for the mean water displacement is shown for the lower middle panel.

Title: Supplementary Movie 6

The di-leucine valve regulates water flow between central and upper cavities of L555I mutant.

Description: (**Upper left panel**) The zoom-in of L555I mutant at the valve (red) shows the side chains of L554 (light magenta), L555I (dark magenta), F431 (dark green) and M549 (light green). (**Upper middle panel**) The spatial water density shows water around the valve and transport part of ABCG2. The map is the average of 3 parallel simulations using trajectories of 150 ns. (**Upper right panel**) The color scale of the spatial water density is shown for the upper middle panel. (**Lower left panel**) The zoom-in of L555I mutant at the valve with the same labelling in the upper left panel shows the water molecule (light blue). (**Lower middle panel**) The mean water displacement shows water dynamics around the valve and the transport path in ABCG2. The map is the average of 3 parallel simulations using trajectories of 150 ns. (**Lower right panel**) The color scale for the mean water displacement is shown for the lower middle panel.

Title: Supplementary Movie 7

The di-leucine valve regulates water flow between central and upper cavities of L555C mutant.

Description: (**Upper left panel**) The zoom-in of L555C mutant at the valve (red) shows the side chains of L554 (light magenta), L555C (dark magenta), F431 (dark green) and M549 (light green). (**Upper middle panel**) The spatial water density shows water around the valve and transport part of ABCG2. The map is the average of 3 parallel simulations using trajectories of 150 ns. (**Upper right panel**) The color scale of the spatial water density is shown for the upper middle panel. (**Lower left panel**) The zoom-in of L555C mutant at the valve with the same labelling in the upper left panel shows the water molecule (light blue). (**Lower middle panel**) The mean water displacement shows water dynamics around the valve and the transport path in ABCG2. The map is the average of 3 parallel simulations

using trajectories of 150 ns. (**Lower right panel**) The color scale for the mean water displacement is shown for the lower middle panel.

Title: Supplementary Movie 8

The di-leucine valve regulates water flow between central and upper cavities of L554A L555A double mutant.

Description: (**Upper left panel**) The zoom-in of L554A L555A double mutant at the valve (red) shows the side chains of L554A (light magenta), L555A (dark magenta), F431 (dark green) and M549 (light green). (**Upper middle panel**) The spatial water density shows water around the valve and transport part of ABCG2. The map is the average of 3 parallel simulations using trajectories of 150 ns. (**Upper right panel**) The color scale of the spatial water density is shown for the upper middle panel. (**Lower left panel**) The zoom-in of L554A L555A double mutant at the valve with the same labelling in the upper left panel shows the water molecule (light blue). (**Lower middle panel**) The mean water displacement shows water dynamics around the valve and the transport path in ABCG2. The map is the average of 3 parallel simulations using trajectories of 150 ns. (**Lower right panel**) The color scale for the mean water displacement is shown for the lower middle panel.

Title: Supplementary Movie 9

The di-leucine valve regulates water flow between central and upper cavities of L554I L555I double mutant.

Description: (**Upper left panel**) The zoom-in of L554I L555I double mutant at the valve (red) shows the side chains of L554I (light magenta), L555I (dark magenta), F431 (dark green) and M549 (light green). (**Upper middle panel**) The spatial water density shows water around the valve and transport part of ABCG2. The map is the average of 3 parallel simulations using trajectories of 150 ns. (**Upper right panel**) The color scale of the spatial water density is shown for the upper middle panel. (**Lower left panel**) The zoom-in of L554I L555I double mutant at the valve with the same labelling in the upper left panel shows the water molecule (light blue). (**Lower middle panel**) The mean water displacement shows water dynamics around the valve and the transport path in ABCG2. The map is the average of 3 parallel simulations using trajectories of 150 ns. (**Lower right panel**) The color scale for the mean water displacement is shown for the lower middle panel.

Title: Supplementary Movie 10

The di-leucine valve regulates water flow between central and upper cavities of L554C L555C double mutant.

Description: (**Upper left panel**) The zoom-in of L554C L555C double mutant at the valve (red) shows the side chains of L554C (light magenta), L555C (dark magenta), F431 (dark green) and M549 (light green). (**Upper middle panel**) The spatial water density shows water around the valve and transport part of ABCG2. The map is the average of 3 parallel simulations using trajectories of 150 ns. (**Upper right panel**) The color scale of the spatial water density is shown for the upper middle panel. (**Lower left panel**) The zoom-in of L554C L555C double mutant at the valve with the same labelling in the upper left panel shows the water molecule (light blue). (**Lower middle panel**) The mean water displacement shows water dynamics around the valve and the transport path in ABCG2. The map is the average of 3 parallel simulations using trajectories of 150 ns. (**Lower right panel**) The color scale for the mean water displacement is shown for the lower middle panel.
